# An explainable AI-driven hybrid feature selection approach for coronary artery disease diagnosis

**DOI:** 10.1038/s41598-026-41712-y

**Published:** 2026-03-25

**Authors:** Tarneem Elemam, Hosam Refaat, Mohamed Makhlouf

**Affiliations:** Information Systems Department, Suez Canal University, Ismailia, 41522 Egypt

**Keywords:** Coronary artery disease, Diagnosis, Classification, Feature selection, Explainable AI, Cardiology, Computational biology and bioinformatics, Mathematics and computing

## Abstract

Coronary artery disease (CAD), where the heart does not get enough oxygen-rich blood due to a buildup of fatty matter, is a leading cause of death worldwide. Since its symptoms may not be recognized until a cardiac attack occurs, its early diagnosis is crucial. In this paper, we introduce the SHAP Optimized Wrapper (SHOW) feature selection algorithm, which works in two steps. First, a SHapley Additive exPlanations (SHAP) method is developed using XGBoost, Random Forest (RF), and Support Vector Machine (SVM) classifiers, to rank the features based on their diagnostic significance. Second, an optimized sequential forward selection wrapper technique is employed, whereby the ranked features are evaluated to select the optimal subset. To validate the algorithm, it is used in seven classifiers to classify three public domain CAD data sets. The classifiers are XGBoost, RF, SVM, Decision Tree (DT), Logistic Regression (LR), K-Nearest Neighbors (KNN), and Multilayer Perceptron (MLP). The data sets are the Z-Alizadeh Sani, Cleveland, and Statlog. Leveraging stratified 10-fold cross-validation and delicate hyperparameter tuning, the results reveal that the SHOW algorithm significantly outperforms 14 state-of-the-art competitive algorithms in terms of accuracy and the number of selected features, while also demonstrating favorable performance in clinically relevant metrics such as sensitivity, specificity, AUC, and F1-score. For example, using the XGBoost classifier, the algorithm selects 14 features (out of 55) from the Z-Alizadeh Sani data set, achieving 93.79% accuracy, 93.98% sensitivity, 89.81% specificity, 0.97 AUC, and 93.98% F1-score; 5 features (out of 13) from the Cleveland data set, achieving 86.52% accuracy, 88.55% sensitivity, 85% specificity, 0.89 AUC, and 84.84% F1-score; and 5 features (out of 13) from the Statlog data set, achieving 87.78% accuracy, 80% sensitivity, 92.67% specificity, 0.90 AUC, and 85.18% F1-score. These figures are not matched by any of the 14 competitive algorithms.

**Supplementary Information:**

The online version contains supplementary material available at 10.1038/s41598-026-41712-y.

## Introduction

Cardiovascular diseases (CVDs) constitute a class of disorders that affect the heart and blood vessels. They include coronary artery disease (CAD), cerebrovascular disease (stroke), peripheral vascular disease, heart failure, rheumatic heart disease (RHD), congenital heart disease, and cardiomyopathies^1^. CVDs represent the primary cause of death worldwide. In a 2021 report, the World Health Organization (WHO) stated that approximately 17.9 million people died from CVDs in 2019. This number constitutes 32% of global deaths, with 85% caused by heart attack and stroke. In addition, among the top 10 global causes of mortality in 2019, CAD, also known as ischemic heart disease, is the leading cause for death^2^. The WHO also estimates that CAD will cause the death of approximately 23.6 million people in 2030^3^.

CAD is a condition that affects the blood vessels responsible for delivering blood to the heart^4^. It occurs when plaque accumulation narrows or blocks the coronary arteries by obstructing the arterial wall, which is called atherosclerosis. Since symptoms may not be recognized until after the cardiac attack, early diagnosis and treatment are crucial for decreasing mortality rates^5^.

Diagnosis of CAD requires specialized devices and trained medical professionals. Common diagnostic methods include blood tests, angiography, and electrocardiography (ECG). Although each of these methods has certain advantages and disadvantages, they are generally costly and may lead to several side adverse. Alarmingly, over 75% of CAD cases are reported in low- and middle-income countries, which makes the CAD testing phase economically unfeasible. As a result, many individuals lose their lives due to CAD at relatively young ages because the diagnosis is not performed in a timely manner^3^.

Over the past decades, significant efforts have been made to develop computer-aided systems in healthcare^6^. Along this line, cost-effective methodologies can be developed to check some physical and biochemical indicators for the diagnosis of CAD. Especially with the continuous advancement of information technology, it has become possible to diagnose CAD by examining certain parameters instead of relying on the results of costly medical devices. As highlighted in a recent review^7^, there are 149 studies focused on diagnosing CAD using ML techniques. Beyond traditional ML approaches, ensemble based ML methods have demonstrated strong effectiveness in disease diagnosis tasks by combining multiple learners to improve robustness and diagnostic performance^8^. More recently, deep learning based approaches have been investigated for CVDs diagnosis. In^9^, a hybrid framework integrating a transformer encoder with a residual convolutional network is employed for classifying heart sound signals obtained from phonocardiography recordings, demonstrating promising performance across multiple classification scenarios.

Medical data sets often contain a large number of irrelevant or/and redundant features. The inclusion of these features causes three major issues in performing the classification task: slowing down the learning process, increasing the risk of overfitting, and degradation of the model’s performance^10^. Therefore, dimensionality reduction (DR) is essential, as it plays a crucial role as a pre-processing step in the classification process.

DR techniques are generally grouped into feature extraction and feature selection approaches. In the feature extraction approach, the aim is to capture the most relevant information from the original data and represent it in a reduced-dimensional space. Commonly used feature extraction techniques include Principal Component Analysis (PCA), t-Distributed Stochastic Neighbor Embedding (t-SNE), Linear Discriminant Analysis (LDA), and Canonical Correlation Analysis (CCA)^11^. In the feature selection approach, the dimensionality of the feature space is reduced by removing unnecessary or redundant features while preserving the model’s predictive performance. Recently, several feature selection methods have emerged, typically classified into four categories: filter, wrapper, embedded, and hybrid^12^.

Filter methods, known for their scalability, operate independently of ML algorithms. They select the most relevant features by evaluating the intrinsic properties of the data based on statistical measures. Though they improve prediction accuracy, they perform poorly compared to wrapper and embedded methods^13^. Some common statistical measures used are Information Gain (IG), Minimum Redundancy Maximum Relevance (mRMR), Relief-F, Correlation-based Feature Selection (CFS), Chi-square (${X}^{2}$), and Symmetrical Uncertainty (SU)^14^. Wrapper methods evaluate various subsets of features by assessing the performance of an ML model. While they often give better results, they require significant computational resources. In addition, they yield merely the optimal feature set, without clarifying the contribution of these features to the model’s behavior^13^. Forward Feature Selection (FFS), Backward Feature Elimination (BFE), and Recursive Feature Elimination (RFE) are among the most commonly used wrapper methods.

*Forward Feature Selection* An iterative approach that begins with an empty model. Initially, each feature is used separately to train the model, and the one that results in the highest classification accuracy is selected. Next, the model is trained using combinations of the selected feature and one additional feature from the remaining set, selecting the pair that achieves the best classification accuracy. This iterative process continues until a subset of features is found that yields superior classification accuracy compared to all other evaluated subsets^15^.

*Backward Feature Elimination* An approach that starts with all available features included in the model. Then, at each iteration, the least important one, determined by specific evaluation criteria, is eliminated. This process is repeated until feature removal no longer improves performance^16^.

*Recursive Feature Elimination* An approach that applies a backward selection process to identify the optimal feature set, where a model is first built using all features, and their importance scores are calculated. Next, features are ranked, and the less significant or redundant ones are iteratively removed according to some model evaluation metrics, continuing until the model contains only the desired number of features^17^.

Embedded methods combine the benefits of both filter and wrapper approaches. They are implemented by ML algorithms that possess their own built-in feature selection methods, and they typically reveal only the magnitude of the feature’s importance, without explaining its specific influence on the model. The most widely used embedded methods include Ridge, and Least Absolute Shrinkage and Selection Operator (LASSO)^18^. Beyond traditional feature selection approaches, Explainable AI (XAI) has emerged as a powerful tool for selecting relevant features.

XAI aims to enhance the interpretability of ML models by providing methods that make their decision-making processes understandable to humans^19^. A common technique in XAI is the identification of feature attributions—scores that quantify how much each input feature contributes to the model’s output. Features with low attribution scores are considered to have minimal influence and can therefore be removed without significantly impacting model performance^20^. XAI methods do not fall into the traditional categories of feature selection. Rather, they represent a separate category of methods, often referred to as interpretability-based methods, and share some similarities with embedded methods where feature importance is derived from a trained ML model. Moreover, XAI methods take into account feature interactions, temporal dependencies, and the ways in which features influence the model^13^. Some widely used XAI methods are SHapley Additive exPlanations (SHAP), Permutation Feature Importance (PFI), and Local Interpretable Model-Agnostic Explanations (LIME)^21^. Since each feature evaluation approach has its own strengths and weaknesses, hybrid methods integrate various types of feature selection methods to leverage their complementary benefits. In many cases, these hybrid methods outperform standalone ones^22^. In this study, we propose a hybrid feature selection method called SHOW.

The main objective of this research is to develop an efficient feature selection approach for CAD diagnosis that achieves high diagnostic accuracy while using a minimal number of features across multiple CAD data sets. This is achieved by integrating an ensemble SHAP-based feature ranking method with an optimized sequential forward selection wrapper technique.

The main contributions of this study are summarized as follows:

- A new two-stage hybrid feature selection algorithm, called SHOW, is proposed. In the first stage, the SHapley Additive exPlanations (SHAP) explainable AI technique is applied to three carefully selected classifiers—chosen based on recent studies for their proven effectiveness in CAD diagnosis—to rank features according to their diagnostic significance. The obtained SHAP-based ranks are then aggregated using an ensemble strategy to produce a more stable and reliable feature ranking while mitigating classifier-specific bias. In the second stage, an optimized sequential forward selection wrapper technique is applied, guided by the SHAP-based feature ranking. Unlike traditional sequential forward selection, which exhaustively evaluates all remaining candidate features at each iteration, the proposed optimized strategy considers only the next highest-ranked feature. The candidate feature is retained only if its inclusion improves the classification performance; otherwise, it is discarded. This strategy significantly reduces redundant evaluations and computational overhead while preserving diagnostic performance.
- To the best of our knowledge, this study is among the first to employ SHAP explicitly as an ensemble based feature ranking tool for CAD diagnosis.

The rest of the article is organized as follows. Section 2 reviews the recent related work. Section 3 describes the proposed CAD diagnosis system. Section 4 details the experimental conditions, obtained results, and comparison with other state-of-the-art studies. Finally, Sect. 5 concludes the article and discusses future research directions.

## Related work

Extensive research has been conducted in recent years on each component of the proposed system. With respect to ML models, many studies have addressed their use in diagnosing CVDs. In^23^, the authors present a thorough survey of various ML algorithms for efficient prediction, diagnosis, and treatment of various heart diseases, highlighting ten algorithms: Multilayer Perceptron (MLP), Random Forest (RF), K-Nearest Neighbors (KNN), J48, Artificial Neural Network (ANN), Support Vector Machine (SVM), Naïve Bayes (NB), Logistic Regression (LR), Decision Tree (DT), and C4.5. They conclude that the ML models with the highest prediction accuracy are ANN, J48, and RF, while the models with the lowest prediction accuracy are KNN, C4.5, DT, and MLP.

In^24^, the performance of five ML models, namely CART, NB, RF, Bagged Trees, and AdaBoost is compared. The findings indicate that RF achieves the best results for predicting CVDs risk. A similar view regarding the superiority of RF is reported in^25^, where the authors use four supervised ML algorithms to train and test the models for CVDs status prediction by considering traditional LR as the baseline model. Their results show that RF significantly outperforms other ML algorithms and LR in terms of performance indices. Another view is presented in^26^, where the authors report that the eXtreme Gradient Boosting (XGBoost) model exhibits superior performance in their experiments comparing XGBoost, RF, LR, and Light Gradient Boosting Machine (LightGBM). In^27^, the authors develop a CVDs prediction model using a two-level stacking framework consisting of base and meta learners. The base level includes ten diverse classifiers, such as instance-based (e.g., KNN), ensembles (e.g., GBM and XGBoost), and probabilistic models (e.g., NB), to exploit their complementary strengths. With MLP as the meta-learner, the stacked classifier demonstrates superior performance compared to traditional ML classifiers.

Additional relevant studies have been conducted in the context of CAD diagnosis using ML. In^28^, six ML classification algorithms, namely SVM, LR, LightGBM, XGBoost, Gaussian Naive Bayes (GNB), and RF algorithm, are employed for developing a CAD risk prediction model. Experimental results demonstrate that the RF algorithm provides better accuracy, followed by SVM and LR. In^29^, LASSO LR and RF are applied to develop a risk prediction model for premature CAD, and the results shows that the RF model achieves better predictive performance and is suitable for clinical practice.

The authors in^30^ use some ML algorithms, including MLP, SVM, LR, J48, RF, KNN, and NB, to provide a diagnostic model for CAD. Based on the compared performance metrics, they demonstrate that SVM and RF are the most effective ML algorithms, but among the algorithms, KNN is the least efficient. This view differs among the authors of^31^, who utilize SVM and XGBoost algorithms to train and test the models. They report a significant difference in accuracy percentage of 4.92% in favor of the XGBoost classifier, highlighting it as a superior and efficient option for CAD diagnosis. This view is also shared in^32^, where the authors conduct a comparative analysis of six ML algorithms—LR, SVM, DT, XGBoost, Bagging, and LightGBM—and conclude that XGBoost is the best-performing model, emphasizing its ability to significantly improve predictive accuracy for coronary infarction.

In^33^, ML algorithms which include SVM, KNN, Random Trees (RT), NB, GBM, and LR are applied to build the predictive models for CAD diagnosis. The results show that the RF model achieves the highest accuracy and AUC-ROC, the NB model achieves the highest specificity, and the SVM model achieves the highest sensitivity. In^34^, out of ten classification models, namely DT, XGBoost, RF, Conditional random forest (Cforest), LR, SVM, ANN, KNN, LDA, and NB, which are evaluated on the CAD data sets, the authors find that the SVM model is the most suitable for predicting the health conditions of CAD patients. In^35^, the performance of an ensemble ML model for CAD prediction is evaluated, and the findings show that it outperforms individual approaches and highlight its potential as a valuable tool for early diagnosis and treatment.

From the above studies, it becomes clear that no agreement exists regarding which ML model performs best for CAD diagnosis. The performance largely depends on several factors, including the data set used for training, the selected features, and the parameter settings.

Recognizing the crucial role of feature selection in enhancing model performance, researchers have devoted considerable attention to this aspect. In^36^, an advanced hybrid ensemble gain ratio feature selection (AHEG-FS) model is introduced, which integrates four techniques, namely ensemble feature selection, BFE, gain ratio feature selection, and an area under the curve (AUC). In addition to accuracy, AUC is employed as an evaluation metric for the proposed feature reduction method. The first two techniques yield subsets of highly significant and top-ranked features, which are subsequently evaluated using nine ML algorithms—AdaBoost, KNN, LR, SVM, classification-via-clustering, Boosted Regression Tree (BRT), Stochastic Gradient Boosting (SGB), RF, and NB. In the third and fourth techniques, AUC values are assessed for the ML algorithms, and BFE is applied to eliminate redundant features. Experiments on four heart disease data sets demonstrate that the proposed model achieves clearly superior performance.

In^37^, the authors first apply ten traditional ML algorithms to the Z-Alizadeh Sani data set, and then select the three best-performing models (nuSVM, nu-SVC, and LinSVM).To further enhance their performance, a new genetic training technique called the New 2-level Genetic optimizer (N2Genetic optimizer) is designed, which integrates cross-validation with Genetic Algorithm (GA) and Particle Swarm Optimization (PSO) for parameter optimization and feature selection. The findings show that the N2Genetic-nuSVM algorithm clearly outperforms other algorithms in CAD diagnosis. In^38^, the authors introduce a hybrid feature selection algorithm named heterogeneous hybrid feature selection (2HFS). They apply the proposed algorithm to four CAD data sets and use RF, DT, XGBoost, and GNB as classifiers. The experimental results prove the efficiency of the proposed algorithm.

In^39^, the authors propose an integrated method using ML, and utilize various ML methods including RTs, DT of C5.0, SVM, and DT of Chi-squared automatic interaction detection (CHAID) for modeling. Through these methods, the predictive features are selected based on the order of their priority. The authors conclude that the RT model outperforms other models. In^40^, the authors present an emotional neural networks (EmNNs) ML model hybridized with conventional PSO technique for the diagnosis of CAD. In addition, they implement four feature selection methods, namely Fisher, Relief-F, mRMR, and Weight by SVM (WBSVM) to boost the functionality of the proposed model. The authors report that the proposed model called PSO-EmNN achieves the best performance when compared with the PSO based adaptive neuro-fuzzy inference system (PSO-ANFIS).

In^41^, a hybrid feature selection methodology named QBSO-FS is introduced, combining the Bee Swarm Optimization (BSO) method with Q-Learning to optimize feature selection for CAD prediction. The Z-Alizadeh Sani, Cleveland, and Statlog data sets are utilized to evaluate the model using various classifiers. Experimental results show that the proposed method yields the optimal feature subset and achieves the highest classification performance with the SVM model. In^4^, the authors present an effective CAD diagnosis method that leverages key clinical features. Their approach applies eight search strategies—evolutionary, greedy stepwise, best first, rank, PSO, harmony, genetic, and multi-objective evolutionary search—for feature selection on the Z-Alizadeh Sani data set, while PCA is applied for feature extraction, and AdaBoostM1 is employed for classification. The experiments conducted demonstrate the effectiveness of their method and highlight its potential as a cost-effective decision-support tool for medical practitioners in CAD diagnosis.

In^42^, the authors analyze the accuracy of the prediction of CAD using Bayes Net, NB, RF, C4.5, MLP, Projective Adaptive Resonance Theory (PART), as well as ensemble ML algorithms such as bagging, boosting, stacking, and majority voting. The experiments are conducted on the Cleveland data set. Comparison of the results shows that majority voting achieves the highest improvement in accuracy, and its performance improves further when applying the brute force feature selection method. In^43^, a Random Forest-Feature Sensitivity and Feature Correlation (RF-FSFC) technique is presented, where the sensitivity-based feature selection process ranks features according to their value in assessing CAD risk, and the feature correlation analysis phase detects the similarity between features. The proposed methodology is applied to the Cleveland data set. Experimental results show that the RF-FSFC method significantly enhances prediction accuracy compared to other methods that do not employ the integrated feature selection strategy.

In^44^, the role of feature selection in optimizing ML algorithms for CAD prediction is examined. The study uses the Cleveland data set and applies sixteen feature selection techniques, grouped into three categories: filter, wrapper, and evolutionary methods. Subsequently, seven ML algorithms—BN, NB, MLP, SVM, RF, LogitBoost, and J48—are employed to identify the most effective models. The findings demonstrate that the highest accuracy is achieved by the SVM model using the CFS/IG/SU filter method. These three filters select the same ten features. In^45^, the authors propose a multi-tier ensemble (MTE) model that incorporates RF-based feature selection to enhance heart disease diagnosis. The framework consists of three tiers, employing bagging, boosting, and stacking ensemble techniques, respectively. To evaluate the model, three data sets are employed, namely Cleveland, Statlog, and a composite data set that integrates five well-known data sets—Cleveland, Statlog, Long Beach VA, Switzerland, and Hungarian. Experimental results reveal that the proposed model outperforms other models. In^46^, the authors propose a hybrid feature selection technique HRFLC (RF + AdaBoost + Pearson Coefficient) for the prediction of CAD. The proposed method is applied to different ML techniques and tested on a data set from the UCI ML Repository. The authors conclude that the HRFLC technique helps in predicting diseases in a very efficient manner, and improves the accuracy level in prediction.

In^3^, several feature selection techniques for CAD diagnosis are evaluated, including $\:{X}^{2}$, gain ratio, IG, Relief-F, Conditional Mutual Information Maximization (CMIM), SVM, BSO, and a domain knowledge (DK)-based method. The authors propose two ensemble-based approaches: an exhaustive ensemble feature selection (EEFS) method and a probabilistic ensemble feature selection (PEFS) method. The former approach is tested on three CAD data sets—Cleveland, Statlog, and Z-Alizadeh Sani—whereas the latter approach is applied only to the Z-Alizadeh Sani data set. Both approaches are evaluated using six classification algorithms and four ensemble voting algorithms. The results indicate that the MLP classifier consistently achieves the highest performance across all three data sets under both approaches.

In^47^, the authors propose a novel binary Artificial Protozoa Optimizer (bAPO) algorithm with eight different V-shaped and S-shaped transfer functions, which are evaluated on fourteen well-known biological data sets. The results show that V-shaped variants generally achieve higher fitness values but select more features, whereas S-shaped variants provide better dimensionality reduction while maintaining consistent classification accuracy. In^48^, the authors develop a Soft Path Feature Selection (SPFS) algorithm that focuses on accumulating soft path costs to identify critical diagnostic indicators for early coronary heart disease (CHD) detection. The algorithm minimizes redundancy while emphasizing feature interactions. When tested on four data sets, it demonstrates strong performance, especially on large and imbalanced data sets, effectively addressing challenges where many existing methods fall short.

Once more, the mentioned studies indicate that no feature selection method can be considered the best across all circumstances. Table 1 summarizes the reviewed studies on CAD diagnosis using feature selection methods presented in this section, highlighting major contributions, advantages, data sets used, and limitations.

**Table 1:** Summary of existing CAD diagnosis studies according to their major contributions, advantages, data sets used, and key limitations.

Study	Major contributions	Advantages	Data sets used	Limitations/Gaps
^37^	Proposed an ML-based CAD diagnosis framework using an N2Genetic Optimizer	- High diagnostic accuracy - Effective feature selection	Z-Alizadeh Sani	- Evaluation is limited to a single data set - Class imbalance is not addressed - Training and testing times are not reported. - Performance evaluation relies mainly on traditional metrics, without incorporating more imbalance-sensitive measures such as AUC or F1-score.
^42^	An ensemble-based CAD diagnosis framework employing a brute-force feature selection method	- Improved diagnostic accuracy compared to single classifiers	Cleveland	- Evaluation is limited to a single data set - Runtime is not reported - Performance evaluation mainly relies on the accuracy metric - Limited emphasis on dimensionality reduction
^38^	A new hybrid feature selection method with class balancing	- Explicit imbalance handling	Z-Alizadeh Sani, Hungarian, and Long-beach-va,	- Training and testing times are not reported - Limited dimensionality reduction, with a relatively large subset of features retained.
^39^	Ranking significant CAD features using a RTs model	- Simple and interpretable feature importance ranking	Z-Alizadeh Sani	- Class imbalance is not addressed - Evaluation is limited to a single data set - Training and testing times are not reported - Limited focus on dimensionality reduction
^40^	A hybrid PSO-based EmNN for CAD diagnosis	- Global optimization capability - Captures nonlinear feature relationships	Z-Alizadeh sani, Cleveland, and Statlog	- There remains scope for further accuracy improvement, particularly on the first data set. - Runtime is not reported - Class imbalance is not addressed
^41^	Hybrid feature selection model combining BSO and Q-learning	- Adaptive and efficient feature selection	Z-Alizadeh sani, Cleveland, Statlog, and Cardiovascular disease data set	- Feature reduction effectiveness may be limited for high-dimensional data sets - Computational complexity increases with parameter tuning - No data balancing technique is applied
^45^	Multi-tier ensemble prediction model for CAD diagnosis	- High diagnostic accuracy achieved through a multi-tier ensemble structure	Statlog and Curated data set	- High model complexity - Limited clinical interpretability due to the ensemble structure - Training and testing times are not reported
^43^	A novel feature selection approach with integrated feature sensitivity and feature correlation	- Reduces feature redundancy through correlation analysis	Cleveland	- Evaluation is limited to a single data set - Training and testing times are not reported - Limited emphasis on dimensionality reduction
^44^	Analysis of multiple feature selection methods on various ML classifiers	- Provides empirical insights into the performance of different feature selection and classifier combinations	Cleveland	- Evaluation is limited to a single data set - No novel method proposed - Limited emphasis on achieving minimal feature subsets, as optimal performance is obtained using relatively large feature sets.
^46^	A novel hybrid feature selection technique combining RF, AdaBoost, and Pearson correlation coefficient	- Reduces feature redundancy	Statlog	- Evaluation is limited to a single data set - Training and testing times are not reported - Limited focus on dimensionality reduction - Limited experimental comparison with state-of-the-art feature selection methods
^3^	Proposed two feature selection methods: EEFS and PEFS	- Robust performance across multiple data sets	Z-Alizadeh sani, Cleveland, and Statlog	- High computational complexity due to evaluating numerous feature subset combinations - Training and testing times are not reported - Class imbalance is not addressed
^4^	Novel CAD diagnosis method based on search, PCA, and AdaBoostM1 technique	- High performance using very few features	Z-Alizadeh sani	- Class imbalance is not addressed - Evaluation is limited to a single data set - Training and testing times are not reported
^47^	bAPO for wrapper feature selection	- Strong global search capability	Multiple biological data sets (e.g., Heart, Epilepsy, Statlog, Colon Tumor, Dermatology, Breast Cancer, Diabetic, Leukemia-3c, Parkinson, HCV, Lymphoma, Glioma, Prostate_G)	- High computational cost
^48^	Feature selection approach based on soft path accumulative cost	- Considers feature redundancy and interactions	Heart, Z-Alizadeh Sani, Cardiovascular, and Coronary heart disease	a. Sensitive to parameter tuning b. Performance evaluation mainly relies on the accuracy metric c. Runtime is not reported

The proposed study is designed to mitigate these limitations by jointly considering comprehensive evaluation metrics, evaluating training and testing times, using multiple benchmark data sets, addressing class imbalance, and effectively reducing features without compromising diagnostic performance.

With respect to using XAI methods, specifically SHAP as a feature selection technique, some studies have been carried out in several fields^13,49–52^, especially in the medical field, but to diagnose diseases other than CAD. In^53^, the authors employ SHAP values to perform feature selection on a medical data set related to Parkinson’s disease. They combine SHAP values with four classifiers, namely RF, LightGBM, deep forest (gcForest), and XGBoost. The experimental results demonstrate that the four models utilizing SHAP-based feature selection outperform those using F-score, analysis of variance (ANOVA), and mutual information (MI) methods in accurately diagnosing Parkinson’s disease. In^54^, the authors use LASSO as well as SHAP feature selection methods to identify the features related to breast cancer detection, and analyze them through a wide range of performance regulations. They conclude that SHAP feature selection demonstrates optimal performance in this analysis. SHAP is also applied by the authors of^55^ to determine the effective features in predicting dementia patient mortality.

Based on the reviewed literature, it is evident that despite their valuable insights and important foundations provided for CAD diagnosis, their reported performance varies considerably due to multiple influencing factors such as type and number of features, sample size, and patient ethnicity within the data sets. Consequently, no internationally recognized standard method for CAD diagnosis has yet been established. To address this gap, the present study proposes a general CAD diagnosis system capable of working effectively with different data sets.

## Materials and methods

**Fig. 1:**
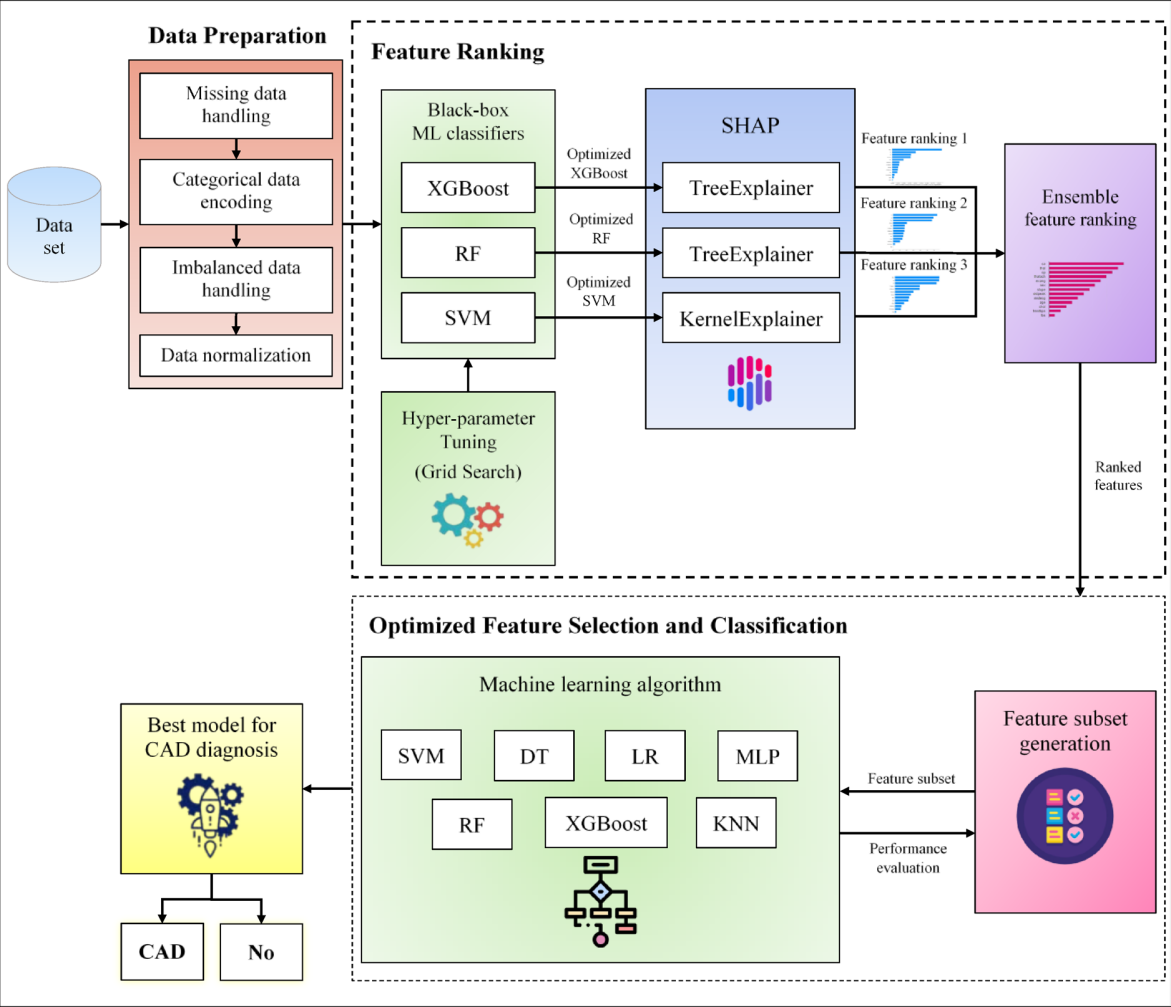
Structure of the proposed CAD diagnosis system.

This section describes the conceptual architecture of the proposed CAD diagnosis system, illustrated in Fig. 1. The system consists of three sequential phases: data preparation, feature ranking, and optimized feature selection with classification. The details of these phases are described below.

### Data preparation

The data preparation phase plays a crucial role in ensuring data quality and preparing it for building the ML model, ultimately enhancing the model’s accuracy and efficiency. This phase involves the following four processes:

#### Missing data handling

Cases with missing values are excluded from the data sets rather than being imputed with synthetic data. Handling missing values is very important as most ML algorithms do not support missing values which can harm the accuracy and dependability of the models^56^.

#### Categorical data encoding

For each categorical feature value within the data set, encoding is performed using label encoding to convert it into a numerical format^57^. Many ML algorithms require numerical input data, and encoding facilitates the incorporation of categorical data into the modeling process without compromising the integrity of information.

#### Imbalanced data handling

To address the issue of class imbalance in the data set, the Synthetic Minority Oversampling Technique (SMOTE) is applied^58^. This method generates synthetic cases of the minority class by interpolating between existing ones and their nearest neighbors. In doing so, it improves the representation of the minority class without simply duplicating existing cases, thereby enhancing model performance. To avoid data leakage, SMOTE is applied after cross-validation splitting and only to the training folds within each iteration.

#### Data normalization

Each value $\:x$ of a feature $\:X$ is normalized using the Min-Max technique^59^. Therefore, each feature value $\:x$ is scaled to a new value $\:{x}_{scaled}$ in the interval [0, 1], according to Eq. (1).

1 $$\:{x}_{scaled}=\frac{x-\mathrm{m}\mathrm{i}\mathrm{n}\left(X\right)}{\mathrm{max}\left(X\right)-\mathrm{m}\mathrm{i}\mathrm{n}\left(X\right)}$$

where min($X$) and max($X$) are the minimum and maximum values of the feature $X$, respectively.

**SHOW algorithm**.

The SHOW feature selection algorithm is designed to enhance the effectiveness of CAD diagnosis by integrating the SHAP technique with an Optimized Wrapper method. The algorithm operates in two key phases:

### Feature ranking

**Fig. 2:**
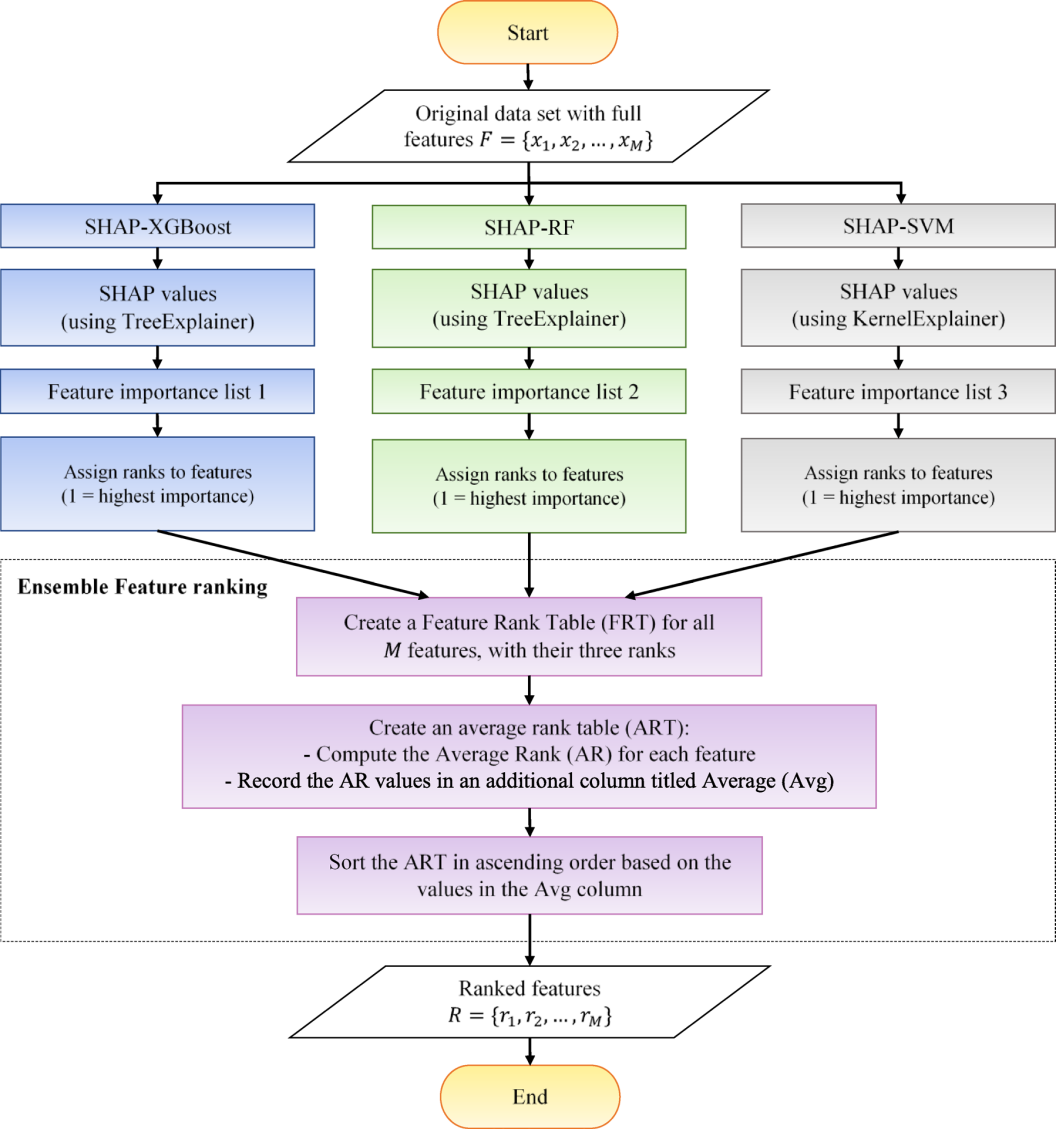
Feature ranking process.

The feature ranking phase aims to prioritize features based on their importance while ensuring transparency and interpretability. To this end, a SHAP-based feature ranking method is adopted. The overall workflow of the feature ranking process is illustrated in Fig. 2. First, the full set of $\:M$ features is used to train three independent classifiers: XGBoost, RF, and SVM. After training each classifier, SHAP values are computed to quantify the contribution of each feature to the model’s predictions. TreeSHAP is applied to the XGBoost and RF models, while KernelSHAP is employed for the SVM model.

For each classifier, SHAP values are calculated for all features across all instances in the data set. The global importance of each feature is then obtained by averaging the absolute SHAP values over all instances. Based on these importance scores, features are sorted in descending order and assigned ranks, where rank 1 corresponds to the most influential feature. This procedure results in three independent ranked feature lists. Finally, to obtain a robust and reliable feature ranking, the three individual ranks are combined using an ensemble strategy by computing the average rank for each feature. The final ranked feature set is obtained by sorting features in ascending order according to their average ranks and is used in subsequent analysis.

#### Classifier selection

In the feature ranking process, three classifiers are employed—XGBoost, RF, and SVM—each using a different allocation mechanism: boosting, bagging, and a hyperplane decision boundary, respectively. These classifiers are chosen for their impressive efficiency in CAD diagnosis, as highlighted in recent studies reviewed in Sect. 2. For simplicity, the SHAP-based ranking approaches are denoted as SHAP-XGBoost, SHAP-RF, and SHAP-SVM. Table 2 summarizes the main advantages and disadvantages of the three adopted classifiers^60,61^.

**Table 2:** Advantages and disadvantages of XGBoost, RF, and SVM classifiers.

	XGBoost	RF	SVM
Advantages	Has features that can help prevent overfitting, such as regularization and early stopping	Able to mitigate overfitting	Able to deal with high-dimensional feature spaces, even with relatively few samples.
	Provides high computational efficiency	Yields high accuracy in many classification tasks.	Provides a trade-off between accuracy and computational cost
	Able to deal with complex data	Less affected by noisy data and outliers in the data set	Able to manage small training data sets effectively
	Can effectively handles missing and imbalanced data	Insensitive to mislabelled training samples	SVM is one of the most memory-efficient methods
Disadvantages	Black-box model	Black-box model	Black-box model
	Requires considerable training time	Can be sensitive to spatial autocorrelation and variations in class proportions	Computationally expensive
	Sensitive to hyperparameters	computationally expensive	Suffers from overfitting risk

#### SHapley additive exPlanations (SHAP)

SHAP, presented by Lundberg and Lee^62^, has gained significant popularity as a method to provide explanations for any ML model. One of its most important contributions is the ability to interpret individual predictions^63^. SHAP assigns Shapley values, which are derived from the principles of cooperative game theory and represent the probabilistic contribution of each player to the final outcome. By formulating the features as players in a coalition game, Shapley values are calculated to distribute the payout fairly. In this context, the players represent the features used in the predictive model. The interaction between features is viewed as a “team” of features, where each feature acts as a team member contributing to the final prediction. Thus, the Shapley value is employed and defined as the average marginal contribution of a feature in an instance among all possible combinations (coalitions) of features^64^.

SHAP provides explanations using a simplified and locally accurate model, represented as a linear function of binary variables. This explanation model can be defined as follows^65^:

2 $$g\left( {x^{\prime } } \right) = \varphi _{0} + \sum\limits_{{i = 1}}^{M} {\varphi _{i} x_{i}^{\prime } }$$

In this formula, $\:g$ is the explanation model, and $\:{x}^{{\prime\:}}\subset\:{\left\{\mathrm{0,1}\right\}}^{M}$ is a coalition vector. $\:M$ is the number of input features in the original instance vector $\:x$. In other words, $\:M$ is the maximal coalition size. The coalition vector $\:{x}^{{\prime\:}}$ represents the presence or absence of each feature in a binary format: a value of 1 means the corresponding feature contributes to the explanation, while a value of 0 means the feature does not contribute. $\:{\varphi\:}_{0}$ is a constant that refers to the base value, which is the average prediction of the original prediction model $\:f$ across all possible instances. The term $\:{\varphi\:}_{i}\in\:\mathbb{R}$ denotes the feature attribution (SHAP value) for the $\:\mathrm{i}$-th feature of a given instance. In other words, $\:{\varphi\:}_{i}$ represents the contribution of $\:\mathrm{i}$-th feature to the model’s prediction for that instance.

**Fig. 3:**
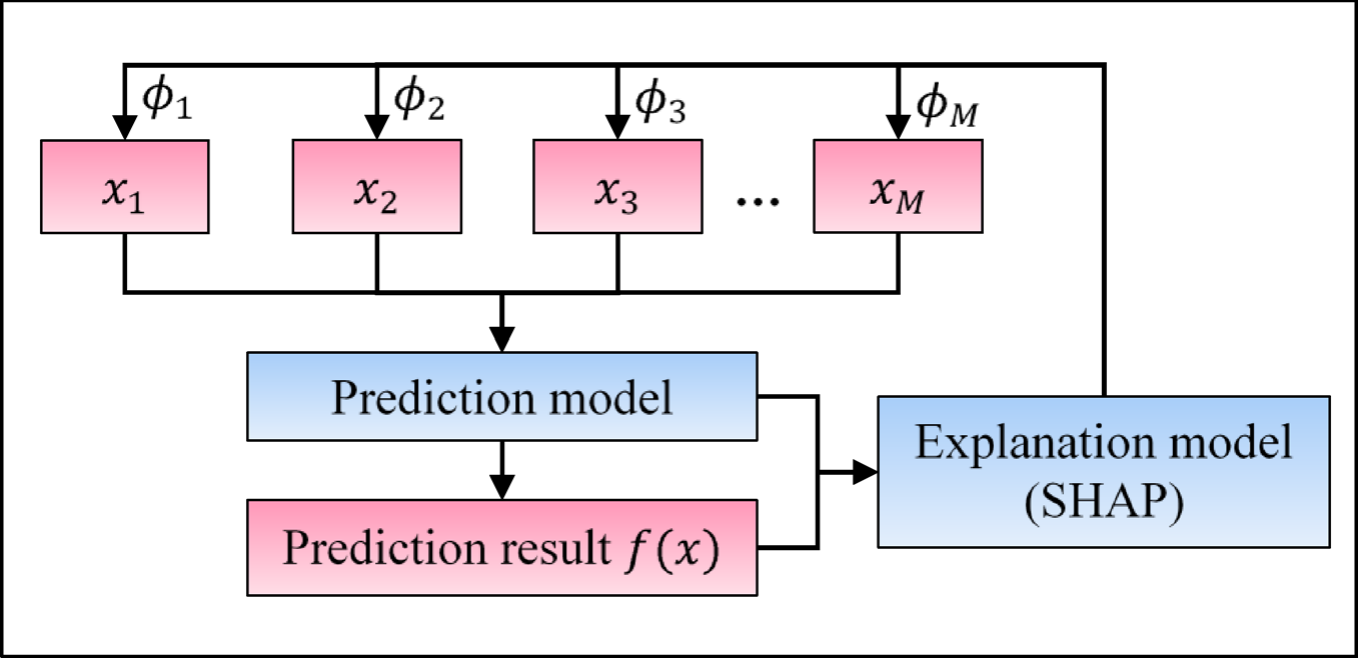
Overview of the relationship between an input instance vector and its prediction using SHAP.

SHAP assigns an attribution effect $\:{\varphi\:}_{i}$ to each feature to reflect its importance for a particular prediction, as illustrated in Fig. 3. The sum of these effects, $\:g\left({x}^{{\prime\:}}\right)$, approximates the output $\:f\left(x\right)$ of the original prediction model; therefore, $\:f\left(x\right)\cong\:g\left({x}^{{\prime\:}}\right)$.

SHAP provides a method to estimate the attribution effect $\:{\varphi\:}_{i}$ of each feature. As expressed in Eq. (3), the SHAP value of a feature $\:{x}_{i}$ is the gain of adding $\:{x}_{i}$, weighted and summed over all possible subsets of features where $\:{x}_{i}$ is not present. Briefly, the SHAP value can be treated as the contribution of $\:{x}_{i}$ to the difference between the actual prediction (including $\:{x}_{i}$) and the average prediction (excluding $\:{x}_{i}$)^66^.

3 $$\varphi _{i} : = \varphi _{i} \left( {f,x} \right) = \frac{1}{{M!}}\sum _{{S \subseteq F\backslash \{ x_{i} \} }} \left[ {\left| S \right|!\left( {M - \left| S \right| - 1} \right)!} \right]\left[ {f(S \cup \{ x_{i} \} ) - f\left( S \right)} \right]$$

where $\:f$ is the original prediction model, $\:x$ is the instance vector, $\:M$ is the total number of input features in $\:x$, $\:{\varphi\:}_{i}$ is the SHAP value corresponding to the feature $\:{x}_{i}$, $\:F$ represents the set of all features used in the model, $\:S$ denotes the coalition (subset) of $\:F$ that does not include the feature $\:{x}_{i}$, and $\:\left|S\right|$ indicates the number of features in subset $\:S$. The term $\:\left|S\right|!$ represents the total number of possible permutations of the features in $\:S$. The term $\:\left(M-\left|S\right|-1\right)!$ denotes the number of possible permutations of the remaining features—that is, those not included in $\:S$ and excluding $\:{x}_{i}$. Together, the product $\:\left|S\right|!\left(M-\left|S\right|-1\right)!$ represents the number of possible permutations of the features in $\:S$ within the full feature set $\:F$. The term $\:M!$ refers to the number of possible permutations of all features in $\:F$.

For each subset S, the formula computes the difference between two predictions: $\:f(S\cup\:\left\{{x}_{i}\right\})$ and $\:f\left(S\right)$. Here, $\:f(S\cup\:\left\{{x}_{i}\right\})$ indicates the model’s prediction when $\:{x}_{i}$ is included along with the features in $\:S$, whereas $\:f\left(S\right)$ indicates the prediction based only on the features in $\:S$. The term $\:[f\left(S\cup\:\left\{{x}_{i}\right\}\right)-f\left(S\right)]$ represents the marginal contribution of $\:{x}_{i}$ to the prediction when added to $\:S$. Therefore, the formula can be rewritten in the following form^67^:

4 $$\begin{aligned} \varphi _{i} : = \varphi _{i} \left( {f,x} \right) = \; & \frac{1}{{{\mathrm{Total}}\:{\text{ permutations}}\:{\text{ of}}\:{\text{ all}}\:{\text{ features}}}} \\ & \sum\limits_{{{\mathrm{coalitions}}\:{\text{ excluding }}\:x_{i} }} {{\mathrm{weight}}*\left[ {{\text{marginal }}\:{\text{contribution }}\:{\text{of }}\:x_{i} \:{\text{ to}}\:{\text{ coalition}}} \right]} \\ \end{aligned}$$

In SHAP, global feature importance can be evaluated as follows: After calculating the SHAP values for each feature across all instances, these values are aggregated across the entire data set to obtain the average absolute SHAP value for each feature. The average SHAP value reflects the global influence of each feature on the model’s predictions, whereas the average absolute SHAP value indicates the global feature importance, regardless of its positive or negative direction. By sorting the features in descending order according to their importance scores, the feature with the highest importance score most significantly influences the model’s predictions. The global feature importance of the $\:i$-th feature, denoted $\:{I}_{i}$, can be calculated using the following formula^63^:

5 $$I_{i} = \frac{1}{n}\sum\limits_{{j = 1}}^{n} {\left| {\varphi _{i}^{{\left( j \right)}} } \right|}$$

where $\:n$ is the number of instances or the sample size, and $\:{\varphi\:}_{i}^{\left(j\right)}$ is the SHAP value of $\:i$-th feature in the $\:j$-th instance.

SHAP provides a model-agnostic explainer called KernelSHAP, along with several model-specific explainers, including TreeSHAP for tree-based models, LinearSHAP for linear models, and DeepSHAP for deep learning models^68^.

In this study, SHAP is employed as a feature ranking tool using three different classifiers, resulting in three SHAP-based feature ranking approaches: SHAP-XGBoost, SHAP-RF, and SHAP-SVM.

- *SHAP-XGBoost* For the XGBoost classifier, TreeSHAP is utilized to compute feature attributions. TreeSHAP is a model-specific explainer optimized for tree-based ensemble models and enables exact and efficient computation of SHAP values by exploiting the tree structure. This makes it particularly suitable for XGBoost models with reduced computational complexity.
- *SHAP-RF* Similarly, TreeSHAP is applied to the RF classifier. Since RF is also a tree-based ensemble model, TreeSHAP provides fast and accurate estimation of feature contributions by aggregating SHAP values across all trees in the forest.
- *SHAP-SVM* For the SVM classifier, KernelSHAP is employed as a model-agnostic explainer. KernelSHAP approximates SHAP values by sampling feature coalitions and fitting a locally weighted linear model. Although computationally more expensive than TreeSHAP, KernelSHAP is suitable for SVM models, which lack an inherent tree structure.

Each SHAP-based method produces a separate list of feature importance scores for the data set under consideration. In each list, features are ranked according to their importance scores, with rank 1 indicating the most influential feature. To obtain a single robust feature ranking, the individual ranks derived from SHAP-XGBoost, SHAP-RF, and SHAP-SVM are aggregated by computing the average rank for each feature, as explained next.

#### Ensemble feature ranking

To improve the robustness and reliability of the feature ranking process, an ensemble approach is adopted instead of relying on a single ranking method. This approach combines the ranks obtained from three SHAP-based feature ranking methods, leveraging their complementary strengths. The ensemble process consists of three key steps:

1. A feature rank table (FRT) is created with $\:M$ rows and four columns, where $\:M$ represents the number of data set features. The first column lists feature names, while the remaining three columns contain the ranks assigned by the three SHAP-based ranking methods.
2. An average rank table (ART) is constructed with five columns. The first column contains feature names, and the next three columns store the ranks of each feature. The average rank (AR) for each feature is then calculated and recorded in the fifth column, called Average (Avg).
3. The ART is sorted in ascending order based on the Avg column. This ensures that the most significant features appear at the top, while the least significant ones are placed at the bottom.

### Optimized feature selection and classification

**Fig. 4:**
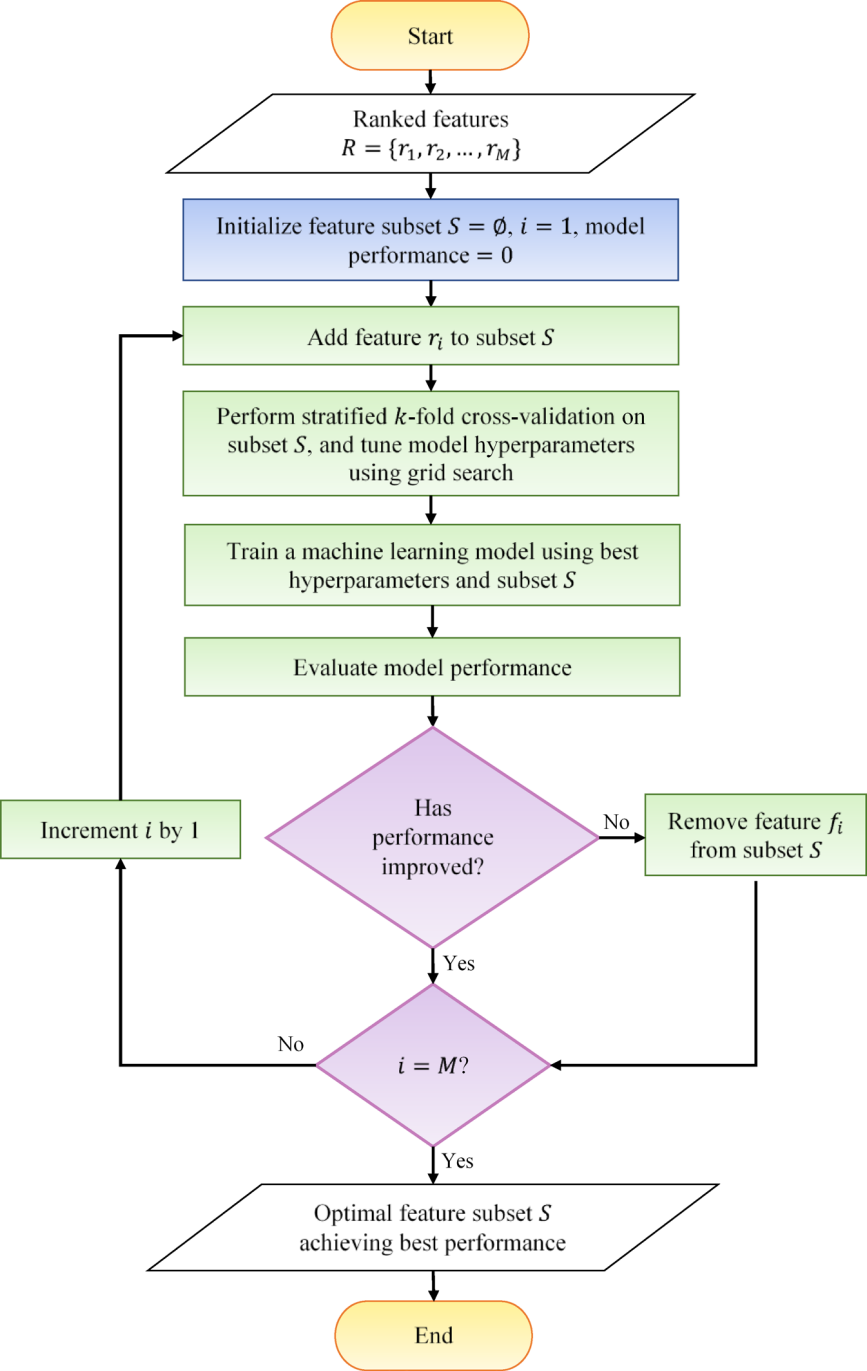
Optimized feature selection and classification process.

Once the features are ranked from highest to lowest importance, the feature selection procedure is applied. The entire optimized feature selection and classification process is illustrated in Fig. 4.

#### Optimized feature selection

Feature selection is a critical aspect of ML, as including irrelevant or redundant features can significantly degrade classifier performance. Selecting the most relevant features from the data set helps reduce training time, minimize overfitting, and enhance overall model performance. Furthermore, working with more informative features aids in facilitating early diagnosis. As outlined in Sect. 1, there are four primary types of feature selection methods: filter, wrapper, embedded, and hybrid methods, as well as XAI-based methods referred to as interpretability-based methods.

In this study, an optimized sequential forward selection wrapper technique is proposed to discover the optimal feature subset, where the term “optimized” denotes a guided and improved version of the traditional sequential forward selection wrapper technique. In this approach, the feature selection process begins by adding the top-ranked feature to an empty subset, followed by building the model using this subset and evaluating its performance. Subsequently, a series of iterative steps is conducted. In each iteration, the next highest-ranked feature is added to the existing subset, the model is built, and its performance is evaluated. If the inclusion of the new feature improves the model’s performance, it is retained in the subset; otherwise, it is removed. At the end of the iterative process, the features that remain in the subset are those that have demonstrated the ability to improve diagnostic accuracy. To avoid bias caused by the initialization order, the importance of the top-ranked feature is validated by examining the effect of removing it from the final selected feature subset.

#### Classification

Classification techniques are employed to assign data instances to a predefined set of classes. In principle, any ML classifier can be used for CAD diagnosis, but in our experiments we focus on seven classifiers, namely XGBoost, RF, SVM, DT, LR, KNN, and MLP. The choice of these classifiers is motivated by recent literature on CAD diagnosis, as outlined in Sect. 2.

- *eXtreme Gradient Boosting (XGBoost)*

XGBoost is an ensemble learning algorithm based on decision trees and applies the gradient boosting approach. The basic principle behind the gradient boosting decision tree (GBDT) method is to generate multiple weak learners, where each new tree is trained to correct the prediction errors made by the previous ones. By combining these weak learners, a strong learner is obtained. In addition, XGBoost provides several advantages, including mechanisms to reduce overfitting, the ability to tune hyperparameters, and support for parallel computation to speed up processing^69^.

- *Random Forest (RF)*

RF is an ensemble learning algorithm that falls under the bagging category of decision tree-based algorithms. It builds multiple decision trees, where each tree is trained on a random subset of features and samples. This randomness reduces the correlation among the trees and helps to mitigate overfitting. RF integrates the classification outputs from all decision trees and determines the final class label according to the majority vote^49^.

- *Support Vector Machine (SVM)*

SVM is a well-established ML algorithm widely applied to both classification and regression problems. It creates a decision boundary (hyperplane) in an M-dimensional feature space to separate data points from different classes. The optimal hyperplane is defined as the one that maximizes the margin—the distance between the hyperplane and the nearest data points from each class, referred to as support vectors. These support vectors determine both the location and direction of the hyperplane^59^.

- *Decision Tree (DT)*

DT is an ML algorithm that builds a classification or regression models in a tree-like structure. The DT model is easy to understand: each internal node corresponds to a test on a specific feature, each branch (edge) represents the outcome of that test, and each leaf node indicates the predicted class label^70^.

- *Logistic Regression (LR)*

LR is a widely used ML algorithm for binary classification tasks. Despite its name, LR is not designed for regression tasks but rather for estimating the likelihood that an input belongs to a particular class. This estimation is performed by applying the sigmoid function, which maps the input features to a probability value between 0 and 1. LR is distinguished by its simplicity, computational efficiency, and ease of interpretation, highlighting its value as an analytical technique in multiple domains, including medical diagnosis^71^. The sigmoid function used in LR is expressed as.

6 $$\:f\left(z\right)=\frac{1}{1+{e}^{-z}}$$

where $\:z$ represents the linear combination of the input features weighted by their individual coefficients (i.e., $\:z={\beta\:}_{0}+{\beta\:}_{1}{x}_{1}+{\beta\:}_{2}{x}_{2}$+…+$\:{\beta\:}_{M}{x}_{M}$).

- *K-Nearest Neighbors (KNN)*

KNN is a simple supervised learning algorithm widely applied to tackle classification and regression problems. It identifies the K-nearest neighbors of a new data point by computing the distance between it and each training instance in the feature space, typically using distance metrics such as Euclidean or Manhattan distance. The predicted class label for a new data point is determined by assigning either the most common class label for classification or the average of the class labels for regression^72^.

- *Multilayer Perceptron (MLP)*

MLP is a type of ANN that consists of an input layer, an output layer, and one or more hidden layers in between. Each layer is composed of nodes (artificial neurons) that are fully connected to the neurons in the adjacent layers. MLP follows a feedforward architecture, meaning that data is transmitted without loops from the input layer to the output layer through the hidden layers. Every node in the network calculates the weighted sum of its inputs, multiplies the result by an activation function, and then sends the output to the next layer. During training, the weights and biases associated with the connections between nodes are adjusted using optimization algorithms such as backpropagation to minimize prediction error. MLPs are widely applied in various tasks, including classification, regression tasks, pattern recognition, image and speech recognition, as well as natural language processing (NLP)^73^.

To achieve optimal performance of an ML model, hyperparameter tuning plays a crucial role. In this study, hyperparameter tuning is performed using grid search, one of the simplest techniques for identifying the best set of hyperparameters. Grid search is a brute-force approach that takes a predefined range of values for each hyperparameter, evaluates the model’s performance across all possible combinations, and ultimately identifies the one that yields the best performance.

To guarantee reliable and credible findings, this study employs a stratified 10-fold cross-validation approach. The data set is divided into 10 approximately equal folds, maintaining the original class distribution within each fold. In each iteration, 9 folds are used for training, while the remaining fold is used for testing. This process is repeated 10 times, such that each fold serves once as the testing set. The model’s performance is evaluated in each iteration, and the average performance across all 10 folds is reported as the final result.

There are many metrics to assess the performance of ML models. In the present study, we use the following metrics: Accuracy, Precision, Sensitivity, Specificity, and F1-score, defined as.

7 $$\:Accuracy = \frac{{TP + TN}}{{TP + TN + FP + FN}}$$

8 $$\:Precision=\frac{TP}{TP+FP}$$

9 $$\:Sensitivity\:\left(or\:Recall\right)=\frac{TP}{TP+FN}$$

10 $$\:Specificity=\frac{TN}{TN+FP}$$

11 $$\:F1\:Score = 2*\frac{{Precision*Recall}}{{Precision + Recall}}$$

where $\:TP$ (True Positives) denotes the number of cases that have CAD and are correctly diagnosed by the model, $\:FP$ (False Positives) refers to the number of cases that do not have CAD and are incorrectly diagnosed by the model, $\:TN$ (True Negatives) indicates the number of cases that do not have CAD and are correctly diagnosed by the model, and $\:FN$ (False Negatives) represents the number of cases that have CAD and are incorrectly diagnosed by the model.

**Algorithm 1:**
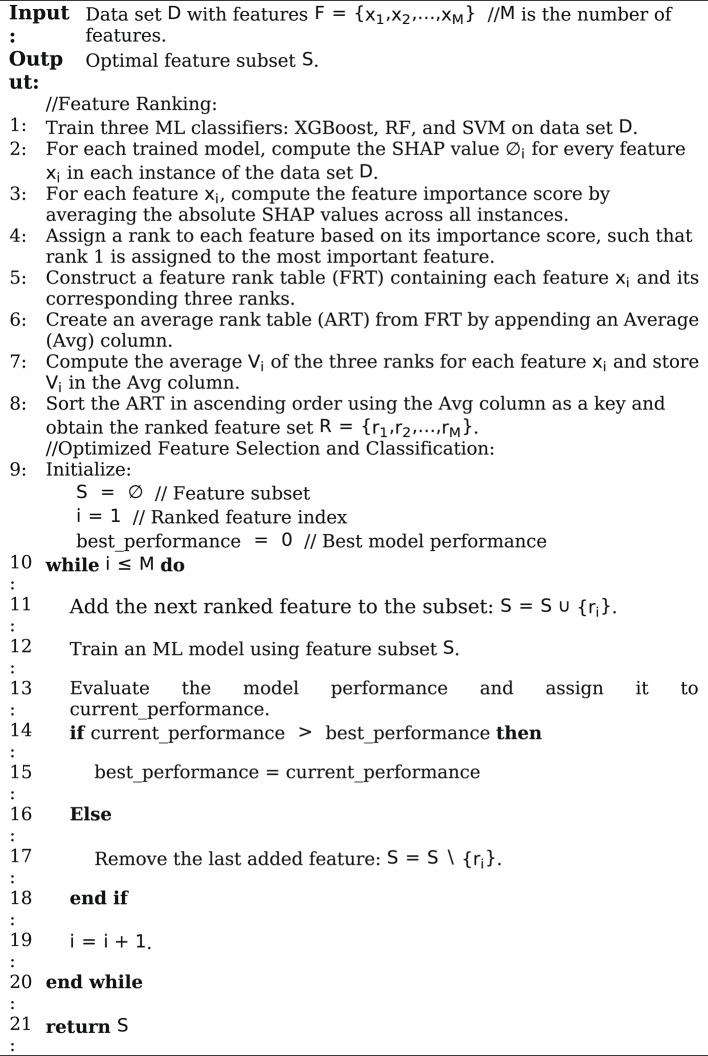
illustrates the pseudocode of the proposed SHOW algorithm.

## Experiments

This section reports the findings of the extensive experiments conducted to validate the proposed SHOW feature selection algorithm. The experimental work was carried out using three CAD data sets, which will be discussed in detail later. All experiments were performed in a Jupyter Notebook (Anaconda3) with Python version 3.11.4 and 64-bit Windows 10 operating system with 8 GB RAM and Intel^®^ Core™ i7-4510U CPU @ 2.00 GHz 2.60 GHz.

### Data sets

The comprehensive review conducted by the authors of^7^ reported that there are 70 distinct CAD data sets available for use in the fields of data mining (DM) and ML. These data sets, collected from three continents, differed in terms of the number of features, their characteristics, patient ethnicity, and sample size. The analysis revealed that larger data sets include fewer features, while medium- and small-sized data sets contain more, and having a small number of features or cases can negatively affect performance, making it difficult to generalize the results. Accordingly, our study evaluated the proposed system using three commonly utilized CAD data sets: Z-Alizadeh Sani, Cleveland, and Statlog. Out of the 70 reviewed data sets, Cleveland is the most frequently cited (3,770 citations), Statlog ranks third (239 citations), while Z-Alizadeh Sani ranks eighth (131 citations). Regarding data completeness, Z-Alizadeh Sani and Statlog are both complete, whereas Cleveland contains six missing values.

Table 3 summarizes the three data sets employed in this study for CAD diagnosis. The Z-Alizadeh Sani data set consists of 303 cases, including 216 CAD cases and 87 normal cases, defined by the presence or absence of ≥ 50% narrowing of the coronary artery diameter on coronary angiography. Each case includes 55 features grouped into four categories: Demographic, Symptom and Examination, ECG, and Laboratory and Eco. Detailed information about these features is presented in Table 4. The Cleveland data set also includes 303 cases, with 139 CAD cases and 164 normal cases, while the Statlog data set includes 270 cases, of which 120 are CAD and 150 are normal. Both Cleveland and Statlog include 13 features, described in Table 5.

**Table 3:** Summary of the three CAD data sets used in this study.

Data set	Number of features	Number of CAD cases	Number of normal cases	Total number of cases
Z-Alizadeh Sani	55	216	87	303
Cleveland	13	139	164	303
Statlog	13	120	150	270

**Table 4:** Description of the features in the Z-Alizadeh Sani data set.

Feature type	Feature	Unit	Description	Feature range	Note
Demographic	Age	years		30, …, 86	
	Weight	kg		48, …, 120	
	Length	cm		140, …, 188	
	Sex		Gender	0, 1	Male (1), female (0)
	BMI	kg/$\:{\mathrm{m}}^{2}$	Body mass index	18, …, 41	
	DM		Diabetes mellitus	0, 1	Yes (1), no (0)
	HTN		Hypertension	0, 1	Yes (1), no (0)
	Current Smoker			0, 1	Yes (1), no (0)
	Ex-Smoker			0, 1	Yes (1), no (0)
	FH		Family history	0, 1	Yes (1), no (0)
	Obesity			0, 1	MBI > 25 (1), otherwise (0)
	CRF		Chronic renal failure	0, 1	Yes (1), no (0)
	CVA		Cerebrovascular accident	0, 1	Yes (1), no (0)
	Airway disease			0, 1	Yes (1), no (0)
	Thyroid disease			0, 1	Yes (1), no (0)
	CHF		Congestive heart failure	0, 1	Yes (1), no (0)
	DLP		Dyslipidaemia	0, 1	Yes (1), no (0)
Symptom and Examination	BP	mm Hg	Blood pressure	90, …, 190	
	PR	ppm	Pulse rate	50, …, 110	
	Edema			0, 1	Yes (1), no (0)
	Weak peripheral pulse			0, 1	Yes (1), no (0)
	Lung Rales			0, 1	Yes (1), no (0)
	Systolic murmur			0, 1	Yes (1), no (0)
	Diastolic murmur			0, 1	Yes (1), no (0)
	Typical chest pain			0, 1	Yes (1), no (0)
	Dyspnea			0, 1	Yes (1), no (0)
	Function class			1, 2, 3, 4	
	Atypical			0, 1	Yes (1), no (0)
	Nonanginal CP		Nonanginal chest pain	0, 1	Yes (1), no (0)
	Exertional CP		Exertional chest pain	0, 1	Yes (1), no (0)
	Low Th Ang		Low threshold angina	0, 1	Yes (1), no (0)
ECG	Q wave			0, 1	Yes (1), no (0)
	ST elevation			0, 1	Yes (1), no (0)
	ST depression			0, 1	Yes (1), no (0)
	Tinversion			0, 1	Yes (1), no (0)
	LVH		Left ventricular hypertrophy	0, 1	Yes (1), no (0)
	Poor R progression		Poor r-wave progression	0, 1	Yes (1), no (0)
	BBB		bundle branch block	1, 2, 3	Normal (1), left (2), right (3)
Laboratory and Echo	FBS	mg/dl	Fasting blood sugar	62, …, 400	
	Cr	mg/dl	Creatine	0.5, …, 2.2	
	TG	mg/dl	Triglyceride	37, …, 1050	
	LDL	mg/dl	Low density lipoprotein	18, …, 232	
	HDL	mg/dl	High density lipoprotein	15, …, 111	
	BUN	mg/dl	Blood urea nitrogen	6, …, 52	
	ESR	mm/h	Erythrocyte sedimentation rate	1, …, 90	
	HB	g/dl	Hemoglobin	8.9, …, 17.6	
	K	mEq/lit	Potassium	3.0, …, 6.6	
	Na	mEq/lit	Sodium	128, …, 156	
	WBC	cells/ml	White blood cell	3700, …, 18,000	
	Lymph	%	Lymphocyte	7, …, 60	
	Neut	%	Neutrophil	32, …, 89	
	PLT	1000/ml	Platelet	25, …, 742	
	EF-TTE	%	Ejection fraction	15, …, 60	
	Region RWMA		Regional wall motion abnormality	0, 1, 2, 3, 4	
	VHD		Valvular heart disease	1, 2, 3, 4	Normal (1), mild (2), moderate (3), Severe (4)

**Table 5:** Description of the features in the Cleveland and statlog data sets.

Feature	Unit	Description	Feature range	Notes
cp		Chest pain type	1, 2, 3, 4	Typical angina (1), atypical angina (2), non-anginal pain (3), asymptomatic (4)
age	year		29, …, 77	
exang		Exercise induced angina	0, 1	Yes (1), no (0)
oldpeak		St depression induced by exercise relative to rest	0, …, 6.2	
ca		Number of major vessels colored by fluoroscopy	0, 1, 2, 3	
sex		Gender	0, 1	Male (1), female (0)
trestbps	mm Hg	Resting blood pressure	94, …, 200	
thalach	bpm	Maximum heart rate achieved	71, …, 202	
fbs		Fasting blood sugar > 120 mg/dl	0, 1	Yes (1), no (0)
restecg		Resting electrocardiographic results	0, 1, 2	
slope		The slope of the peak exercise st segment	1, 2, 3	
chol	mg/dl	Serum cholesterol	126, …, 564	
thal		Results of nuclear stress test	3, 6, 7	Normal (3), fixed defect (6), reversible defect (7)

As reported in the study^3^, which analyzed the three data sets employed, the Z-Alizadeh Sani data set exhibits greater linearity compared with Cleveland and Statlog, whereas Cleveland displays more non-linear behavior. In terms of complexity, Statlog is regarded as the most complex among them. With respect to class distribution, Cleveland and Statlog are relatively balanced, while Z-Alizadeh Sani is imbalanced. The study also emphasized that Z-Alizadeh Sani is sparser than both Cleveland and Statlog. Hence, it is evident that the three data sets differ in their characteristics.

### Results and discussion

The methodology described in Sect. 3 was adopted in developing the proposed system. After pre-processing the data set, the SHAP technique was applied to three classifiers in order to rank the features according to their importance. Subsequently, during the training and testing of seven ML classifiers, an optimized sequential forward selection wrapper was employed with each classifier to identify the optimal feature subset. This process ultimately determined the model that achieved the highest performance using the fewest features.

This section presents the results of the feature ranking process, along with the classification performance of models built using the feature subsets selected by the proposed algorithm. In addition, the classification performance of models utilizing all features is reported. The impact of feature selection on training and testing efficiency is also analyzed. Finally, a comparison between the proposed algorithm and fourteen existing studies is conducted in terms of the number of selected features and the achieved classification accuracy.

#### Results of feature ranking process

**Fig. 5:**
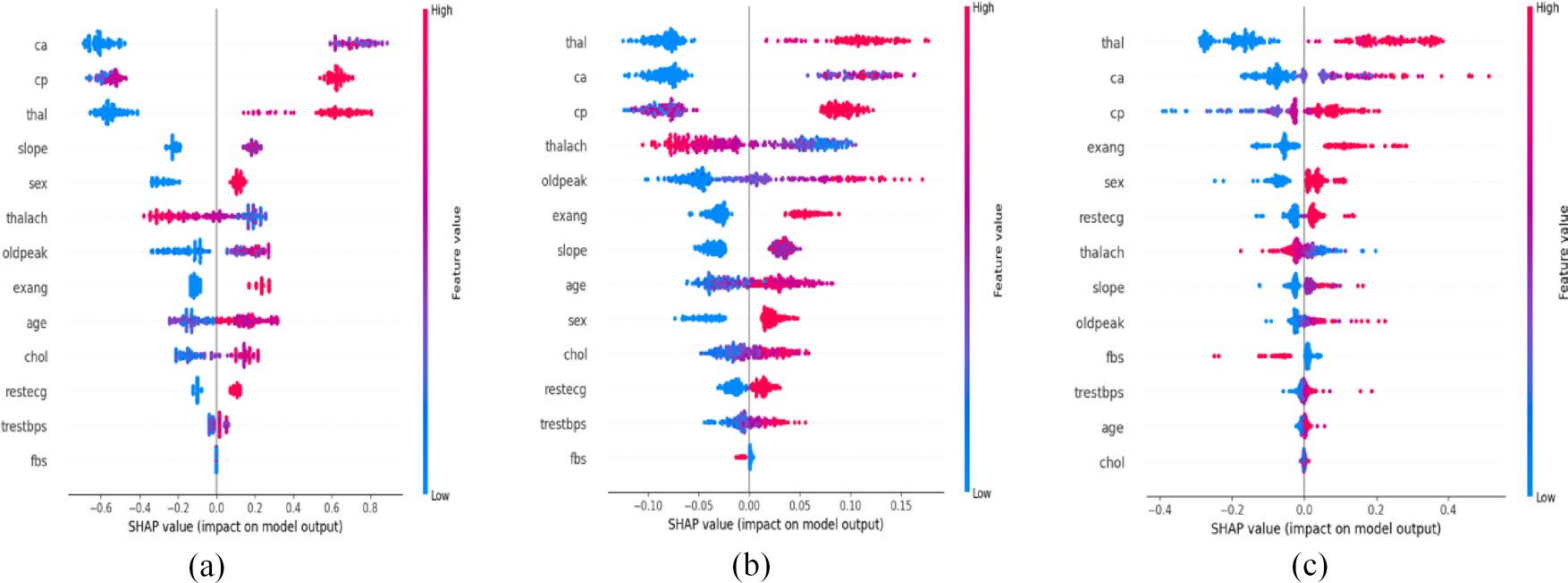
SHAP beeswarm plots for the Cleveland data set using three feature ranking methods: (**a**) SHAP-XGBoost, (**b**) SHAP-RF, (**c**) SHAP-SVM. The y-axis lists the features, and each dot corresponds to one instance. The position of each dot on the x-axis reflects the SHAP value for that instance, capturing both the direction and magnitude of the feature’s contribution to the prediction, while the color reflects the feature value (red = high, blue = low).

Figure 5 shows three beeswarm plots, each corresponding to one of the SHAP-based feature ranking methods: SHAP-XGBoost, SHAP-RF, and SHAP-SVM, respectively. Each plot illustrates the distribution of SHAP values for individual features across instances in the Cleveland data set. The beeswarm plots for the Z-Alizadeh Sani data set and the Statlog data set can be found in Supplementary Figures S1 and S2, respectively.

**Fig. 6:**
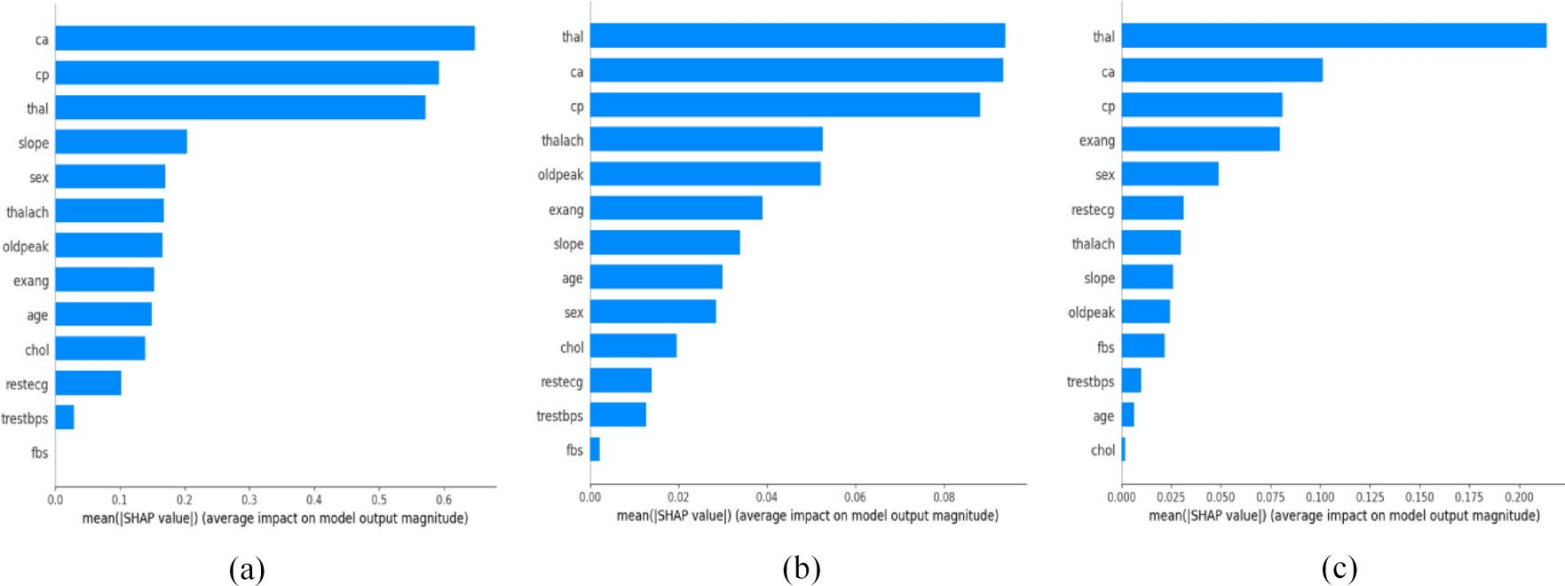
Feature importance bar plots for the Cleveland data set, evaluated using three SHAP-based feature ranking methods: (**a**) SHAP-XGBoost, (**b**) SHAP-RF, and (**c**) SHAP-SVM. Each bar represents the relative importance of individual features in predicting outcomes. The height of each bar reflects the average absolute SHAP values of that feature across all instances, highlighting its average impact on the model.

Figure 6 displays three bar plots, each corresponding to one of the SHAP-based feature ranking methods: SHAP-XGBoost, SHAP-RF, and SHAP-SVM, respectively. Each plot visualizes feature importance for the Cleveland data set, with features ranked based on their contribution to the model’s predictions. Bar plots for the Z-Alizadeh Sani data set and the Statlog data set can be found in Supplementary Figures S3 and S4, respectively. For the Cleveland data set, Tables 6, 7 and 8 report the feature scores and corresponding ranks for the SHAP-XGBoost, SHAP-RF, and SHAP-SVM methods, respectively. The analysis shows that the three methods assign different ranks to the same feature. For instance, SHAP-XGBoost identifies ‘ca’ as the most important feature, whereas SHAP-SVM and SHAP-RF give the highest importance to ‘thal’. In addition, SHAP-XGBoost and SHAP-RF rank ‘fbs’ as the least important feature, while SHAP-SVM places ‘chol’ at the lowest rank. Similar variations are observed for the Z-Alizadeh Sani and Statlog data sets, as detailed in Supplementary Tables S1–S6. To address this variation, the ensemble method outlined in Sect. 3 is utilized to yield an overall rank for each feature based on the collective view of the three SHAP-based feature ranking methods.

**Table 6:** Feature importance scores for the Cleveland data set using SHAP-XGBoost method.

Feature	Feature score	Feature rank
ca	0.647798	1
cp	0.592459	2
thal	0.571232	3
slope	0.203398	4
sex	0.169562	5
thalach	0.167869	6
oldpeak	0.165642	7
exang	0.152477	8
age	0.148538	9
chol	0.139417	10
restecg	0.102270	11
trestbps	0.029267	12
fbs	0.000000	13

**Table 7:** Feature importance scores for the Cleveland data set using SHAP-RF method.

Feature	Feature score	Feature rank
thal	0.093892	1
ca	0.093366	2
cp	0.088299	3
thalach	0.052601	4
oldpeak	0.052163	5
exang	0.038903	6
slope	0.034009	7
age	0.029984	8
sex	0.028474	9
chol	0.019637	10
restecg	0.013983	11
trestbps	0.012699	12
fbs	0.002243	13

**Table 8:** Feature importance scores for the Cleveland data set using SHAP-SVM method.

Feature	Feature score	Feature rank
thal	0.214020	1
ca	0.101312	2
cp	0.080932	3
exang	0.079547	4
sex	0.048741	5
restecg	0.031259	6
thalach	0.030020	7
slope	0.025959	8
oldpeak	0.024318	9
fbs	0.021790	10
trestbps	0.009840	11
age	0.006175	12
chol	0.001681	13

For each data set, a feature rank table (FRT) is constructed after determining the rank of each feature using each of the three SHAP-based feature ranking methods. Table 9 represents the FRT for the Cleveland data set, while the FRTs for the Z-Alizadeh Sani and Statlog data sets are presented in Supplementary Tables S7 and S9, respectively. Additionally, an average rank table (ART) is created, displaying the features sorted from most to least significant based on their average rank (AR) values. Table 10 represents the ART for the Cleveland data set, while the ARTs for the Z-Alizadeh Sani and Statlog data sets can be found in Supplementary Tables S8 and S10, respectively.

**Table 9:** Feature rank table (FRT) for the Cleveland data set.

Feature	SHAP-XGBoost	SHAP-RF	SHAP-SVM
age	9	8	12
sex	5	9	5
cp	2	3	3
trestbps	12	12	11
chol	10	10	13
fbs	13	13	10
restecg	11	11	6
thalach	6	4	7
exang	8	6	4
oldpeak	7	5	9
slope	4	7	8
ca	1	2	2
thal	3	1	1

**Table 10:** Average rank table (ART) for the Cleveland data set.

Feature	SHAP-XGBoost	SHAP-RF	SHAP-SVM	Avg
ca	1	2	2	1.666667
thal	3	1	1	1.666667
cp	2	3	3	2.666667
thalach	6	4	7	5.666667
exang	8	6	4	6.000000
sex	5	9	5	6.333333
slope	4	7	8	6.333333
oldpeak	7	5	9	7.000000
restecg	11	11	6	9.333333
age	9	8	12	9.666667
chol	10	10	13	11.000000
trestbps	12	12	11	11.666667
fbs	13	13	10	12.000000

#### Results of optimized feature selection and classification

Seven ML classifiers were investigated for CAD diagnosis, namely XGBoost, RF, SVM, DT, LR, KNN, and MLP. For each data set, multiple experiments were conducted to assess the performance of the ML models: one experiment employed all available features, while the other experiments employed the features ranked according to their average rank (as per the proposed approach) to determine which feature subset yields the best performance.

To evaluate the performance of each ML model, stratified 10-fold cross-validation was employed, where 90% of the data set cases were used for training and 10% for testing in each iteration. Performance metrics were computed for each fold using Eqs. (7–11). The results for each performance metric were averaged across the 10 folds. The performance of the models, evaluated with the full feature set as well as the subsets identified by the proposed SHOW algorithm, is summarized below.

- *Model performance with full feature set*

Table 11 reports the performance results of the seven ML classifiers using all features for each data set. As shown in the table, XGBoost achieves the best performance on the Z-Alizadeh Sani, Cleveland, and Statlog data sets. The highest accuracy scores are 93.30%, 86.52%, and 87.41% for the Z-Alizadeh Sani, Cleveland, and Statlog data sets, respectively.

**Table 11:** Performance comparison of seven classifiers on three CAD data sets using all features.

Data set	Classifier	Accuracy (%)	Precision (%)	Sensitivity (%)	Specificity (%)	F1-score (%)
Z-Alizadeh Sani (55 features)	XGBoost	93.30 ± 5.89	94.35 ± 8.50	93.01 ± 4.34	88.90 ± 11.88	93.47 ± 5.31
	RF	93.10 ± 5.84	93.70 ± 8.65	93.51 ± 4.30	91.19 ± 10.64	93.34 ± 5.16
	SVM	91.22 ± 5.13	93.27 ± 8.00	89.78 ± 5.06	87.90 ± 13.44	91.22 ± 4.70
	LR	90.98 ± 5.22	92.05 ± 8.91	91.17 ± 5.28	90.71 ± 10.97	91.21 ± 4.51
	MLP	90.76 ± 5.46	92.97 ± 9.62	89.78 ± 4.59	92.16 ± 11.04	90.93 ± 4.65
	KNN	85.21 ± 4.86	94.96 ± 7.72	74.98 ± 6.69	92.14 ± 11.82	83.49 ± 5.23
	DT	83.56 ± 4.23	89.00 ± 7.20	77.64 ± 8.71	86.10 ± 9.45	82.35 ± 5.18
Cleveland (13 features)	XGBoost	86.52 ± 5.70	88.72 ± 5.90	80.93 ± 10.19	89.38 ± 6.87	84.42 ± 7.14
	MLP	84.85 ± 6.61	86.74 ± 9.33	80.16 ± 9.02	88.12 ± 11.34	82.96 ± 7.41
	RF	84.51 ± 5.88	87.48 ± 7.82	78.02 ± 10.45	89.38 ± 8.41	82.06 ± 7.23
	SVM	84.51 ± 6.82	86.41 ± 8.31	79.40 ± 11.21	89.38 ± 8.86	82.30 ± 8.37
	LR	84.51 ± 6.25	87.14 ± 8.20	78.74 ± 10.21	86.25 ± 10.75	82.25 ± 7.27
	KNN	83.51 ± 4.64	85.04 ± 8.92	79.45 ± 9.02	85.00 ± 11.92	81.54 ± 5.45
	DT	79.80 ± 7.89	78.85 ± 9.53	77.36 ± 8.10	75.62 ± 15.92	78.01 ± 8.35
Statlog (13 features)	XGBoost	87.41 ± 6.67	86.97 ± 8.00	85.00 ± 11.06	83.33 ± 8.56	85.53 ± 7.89
	RF	86.30 ± 6.64	90.81 ± 9.30	78.33 ± 13.54	88.00 ± 7.18	83.12 ± 8.58
	MLP	85.56 ± 4.74	87.03 ± 8.42	80.83 ± 11.33	89.33 ± 9.04	82.93 ± 5.75
	LR	85.19 ± 4.68	85.57 ± 7.44	81.67 ± 12.25	89.33 ± 7.42	82.69 ± 6.25
	SVM	84.81 ± 5.09	86.50 ± 8.37	80.00 ± 12.47	88.00 ± 8.84	82.08 ± 6.35
	KNN	83.70 ± 6.02	86.51 ± 8.70	76.67 ± 12.25	92.67 ± 8.14	80.40 ± 7.45
	DT	79.26 ± 7.07	78.67 ± 9.53	74.17 ± 9.46	89.33 ± 7.42	76.02 ± 8.28

- *Model performance with features selected by the proposed SHOW algorithm*

Table 12 reports the performance of the seven ML classifiers for each data set, using the feature subsets obtained through the SHOW algorithm and listed in Tables 13, 14 and 15. Among the classifiers evaluated on the Z-Alizadeh Sani, Cleveland, and Statlog data sets, XGBoost consistently achieved the best performance. On the Z-Alizadeh Sani data set, XGBoost attained an accuracy of 93.79%, precision of 94.39%, sensitivity of 93.98%, specificity of 89.81%, and an F1-score of 93.98% using 14 features. On the Cleveland data set, it achieved 86.52% accuracy, 88.55% precision, 82.36% sensitivity, 85% specificity, and an F1-score of 84.84% using only 5 features. On the Statlog data set, the classifier reached 87.78% accuracy, 92.33% precision, 80.00% sensitivity, 92.67% specificity, and an F1-score of 85.18%, also with 5 features. The key hyperparameter values for the top-performing classifiers are provided in Table 16.

**Table 12:** Performance comparison of seven classifiers on three CAD data sets using the SHOW algorithm.

Data set	Classifier	Number of selected features	Accuracy (%)	Precision (%)	Sensitivity (%)	Specificity (%)	F1-score (%)
Z-Alizadeh Sani	XGBoost	14	93.79 ± 5.49	94.39 ± 8.00	93.98 ± 4.15	89.81 ± 10.73	93.98 ± 4.99
	MLP	10	93.09 ± 5.41	94.27 ± 7.82	92.55 ± 4.75	93.55 ± 9.63	93.18 ± 4.94
	LR	13	93.31 ± 4.25	93.57 ± 7.28	93.94 ± 4.74	90.26 ± 10.41	93.47 ± 3.82
	RF	14	92.85 ± 4.74	95.12 ± 7.54	91.10 ± 5.82	92.12 ± 10.10	92.77 ± 4.51
	SVM	13	92.84 ± 3.75	94.47 ± 6.55	91.60 ± 4.64	91.21 ± 12.30	92.80 ± 3.57
	KNN	13	91.93 ± 5.88	94.21 ± 7.27	89.81 ± 6.82	92.60 ± 12.03	91.77 ± 5.82
	DT	5	90.53 ± 5.06	93.93 ± 8.18	87.81 ± 8.06	87.42 ± 11.40	90.25 ± 4.96
Cleveland	XGBoost	5	86.52 ± 5.07	88.55 ± 8.51	88.55 ± 8.94	85.00 ± 8.93	84.84 ± 5.70
	MLP	7	86.17 ± 5.81	89.52 ± 8.14	80.11 ± 10.26	91.25 ± 8.48	84.06 ± 6.99
	SVM	10	85.85 ± 6.88	86.95 ± 7.50	81.56 ± 10.73	89.38 ± 8.86	83.95 ± 8.16
	RF	6	85.83 ± 5.47	87.70 ± 8.67	81.54 ± 9.24	86.88 ± 9.46	84.02 ± 6.19
	DT	5	85.48 ± 5.54	89.22 ± 7.57	78.57 ± 11.09	80.62 ± 9.46	82.99 ± 6.93
	LR	5	85.17 ± 5.04	89.81 ± 9.36	77.91 ± 9.60	92.50 ± 9.19	82.77 ± 5.69
	KNN	3	85.15 ± 5.36	87.46 ± 8.57	80.11 ± 9.59	86.25 ± 9.19	83.12 ± 6.18
Statlog	XGBoost	5	87.78 ± 2.89	92.33 ± 6.72	80.00 ± 8.50	92.67 ± 6.96	85.18 ± 4.08
	DT	3	86.67 ± 5.79	89.29 ± 10.66	81.67 ± 9.72	90.00 ± 11.64	84.51 ± 6.31
	RF	4	86.67 ± 5.79	89.29 ± 10.66	81.67 ± 9.72	90.67 ± 7.42	84.51 ± 6.31
	MLP	6	86.67 ± 4.74	88.32 ± 8.03	81.67 ± 7.26	90.00 ± 11.25	84.49 ± 5.40
	LR	7	86.30 ± 5.51	90.68 ± 9.99	79.17 ± 10.70	84.67 ± 12.31	83.57 ± 6.39
	SVM	3	85.93 ± 5.93	88.40 ± 11.03	80.83 ± 9.90	86.67 ± 7.30	83.64 ± 6.48
	KNN	4	85.93 ± 6.79	86.31 ± 9.70	82.50 ± 9.46	87.33 ± 10.09	83.91 ± 7.66

**Table 13:** List of features selected for each classifier using the SHOW algorithm on the Z-Alizadeh Sani data set.

Classifier	Selected features
XGBoost	Age, BMI, DM, HTN, Obesity, BP, Typical Chest Pain, Tinversion, CR, TG, LDL, K, EF-TTE, Region RWMA
MLP	Age, Sex, BMI, DM, PR, Typical Chest Pain, Dyspnea, Tinversion, EF-TTE, Region RWMA
LR	Age, HTN, Current Smoker, PR, Edema, Lung rales, Typical Chest Pain, Atypical, Tinversion, Poor R Progression, TG, ESR, Region RWMA
RF	Age, Sex, DM, PR, Systolic Murmur, Typical Chest Pain, Poor R Progression, FBS, CR, TG, LDL, ESR, EF-TTE, Region RWMA
SVM	Age, HTN, Current Smoker, Typical Chest Pain, Nonanginal, St Depression, Poor R Progression, FBS, CR, HB, Neut, PLT, Region RWMA
KNN	Age, BMI, HTN, Current Smoker, Edema, Typical Chest Pain, Nonanginal, St Depression, Tinversion, CR, TG, EF-TTE, Region RWMA
DT	Age, DM, Typical Chest Pain, K, Region RWMA

**Table 14:** List of features selected for each classifier using the SHOW algorithm on the Cleveland data set.

Classifier	Selected features
XGBoost	cp, thalach, exang, ca, thal
MLP	age, cp, thalach, exang, slope, ca, thal
SVM	sex, cp, trestbps, chol, restecg, thalach, exang, oldpeak, ca, thal
RF	cp., trestbps, chol, exang, ca., thal
DT	sex, cp, slope, ca, thal
LR	cp, thalach, exang, ca, thal
KNN	cp, ca, thal

**Table 15:** List of features selected for each classifier using the SHOW algorithm on the Statlog data set.

Classifier	Selected features
XGBoost	sex, cp, slope, ca, thal
DT	cp, ca, thal
RF	cp, chol, ca, thal
MLP	age, cp, exang, slope, ca, thal
LR	cp, chol, thalach, exang, oldpeak, ca, thal
SVM	cp, ca, thal
KNN	cp, chol, ca, thal

**Table 16:** Key hyperparameter values that yield the highest model performance.

Classifier	Z-Alizadeh Sani	Cleveland	Statlog
XGBoost	max_depth = 10, n_estimators = 50, learning_rate = 1.0	max_depth = 5, n_estimators = 40, learning_rate = 0.5	max_depth = 5, n_estimators = 20, learning_rate = 1.0
RF	n_estimators = 150, max_depth = 10, max_features = log2	n_estimators = 50, max_depth = 5, max_features = log2	n_estimators = 50, max_depth = 5, max_features = log2
SVM	C = 30, gamma = 0.1, kernel = rbf	C = 20, gamma = 0.1, kernel = rbf	C = 5, gamma = 10, kernel = rbf
DT	max_depth = 5, criterion = gini	max_depth = 5, criterion = entropy	max_depth = 10, criterion = entropy
LR	C = 100, penalty = l2, solver = lbfgs	C = 0.1, penalty = l2, solver = sag	C = 0.1, penalty = l2, solver = saga
KNN	n_neighbors = 5, metric = euclidean	n_neighbors = 11, metric = euclidean	n_neighbors = 11, metric = manhattan
MLP	activation = relu, hidden_layer_sizes = (120, 80), max_iter = 1000	activation = logistic, hidden_layer_sizes = (120, 80), max_iter = 100	activation = tanh, hidden_layer_sizes = (100, 50), max_iter = 50

The optimal hyperparameter values reported in Table 16 provide additional insight into the behavior of the evaluated classifiers. In general, tree-based models such as XGBoost and RF favored moderate tree depths and a moderate number of estimators, indicating that the selected feature subsets capture sufficient discriminative information without requiring overly complex tree structures. For SVM, the consistent selection of the RBF kernel reflects the non-linear separability of CAD data, while the tuned C and gamma values balance margin maximization and classification accuracy. For neural models, the MLP classifier adopted larger hidden-layer sizes and higher iteration limits when trained on selected features, which may lead to increased model complexity in certain cases. Overall, these tuned hyperparameters align well with the observed performance and efficiency results, confirming that the proposed SHOW algorithm enables effective learning with reduced yet informative feature subsets.

**Fig. 7:**
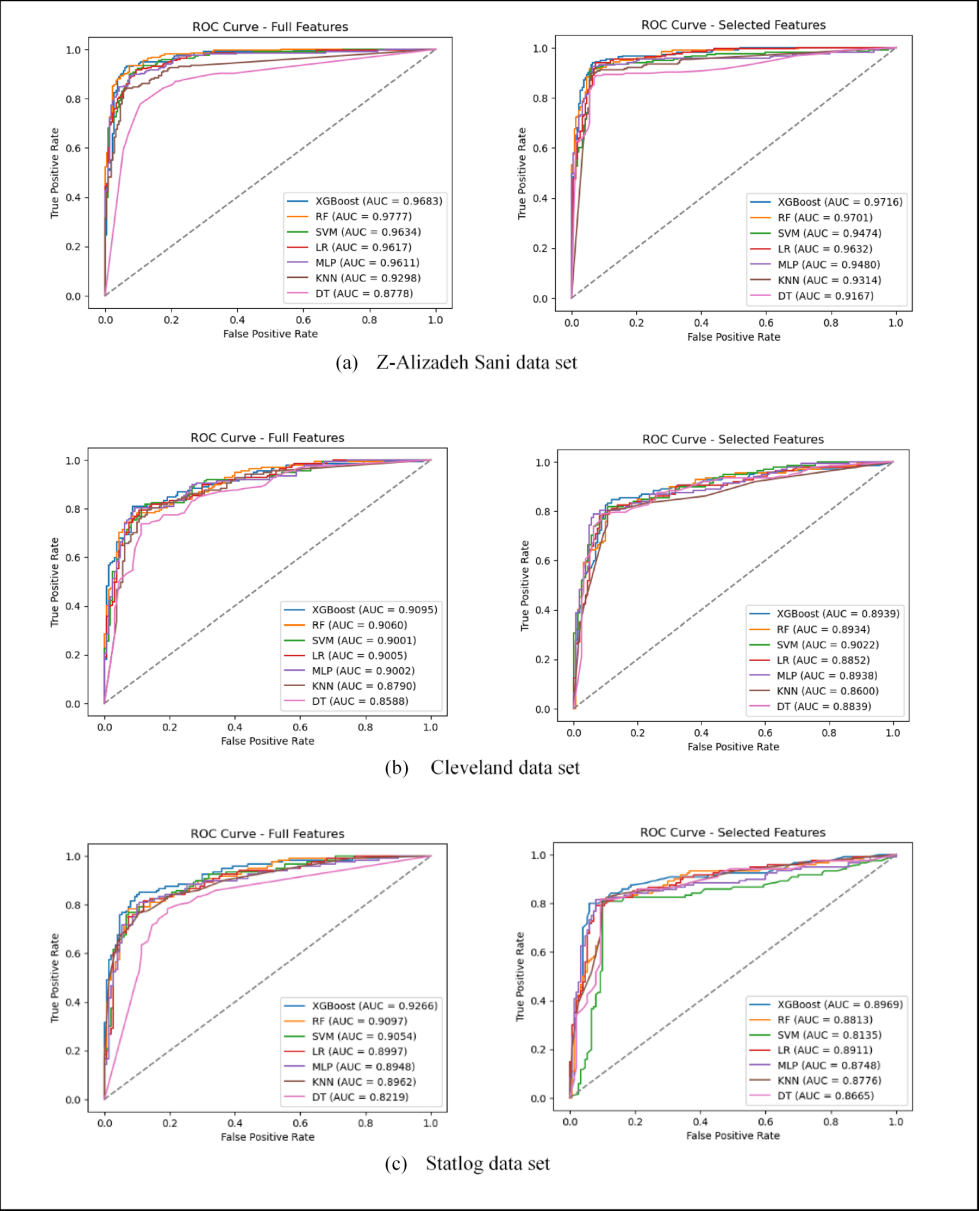
ROC curve comparison of seven classifiers on three CAD data sets: (**a**) Z-Alizadeh Sani, (**b**) Cleveland, and (**c**) Statlog, using full features and selected feature subsets obtained by the SHOW algorithm. The AUC scores for each model are provided in the legends.

The performance of ML models is also evaluated using ROC curves and AUC scores across the three benchmark data sets for both full and selected feature sets, as illustrated in Fig. 7. The curves demonstrate that ensemble-based models, particularly XGBoost and RF, consistently achieve higher AUC values compared to other classifiers, with peak performance reaching up to 0.97 on the Z-Alizadeh Sani data set. The noticeable overlap between the ROC curves obtained using full features and those based on the subsets selected by the SHOW algorithm indicates that the proposed method effectively preserves the most discriminative clinical features while reducing the dimensionality. Although a slight degradation in AUC is observed for some classifiers after feature selection, this reduction is expected due to substantial dimensionality reduction and the removal of marginally informative features; however, the AUC values remain within a competitive range across all data sets, indicating a favorable trade-off between diagnostic performance and model simplicity. In contrast, simpler models such as DT generally exhibit lower AUC scores, highlighting the necessity of ensemble-based approaches for complex CAD diagnosis tasks.

**Fig. 8:**
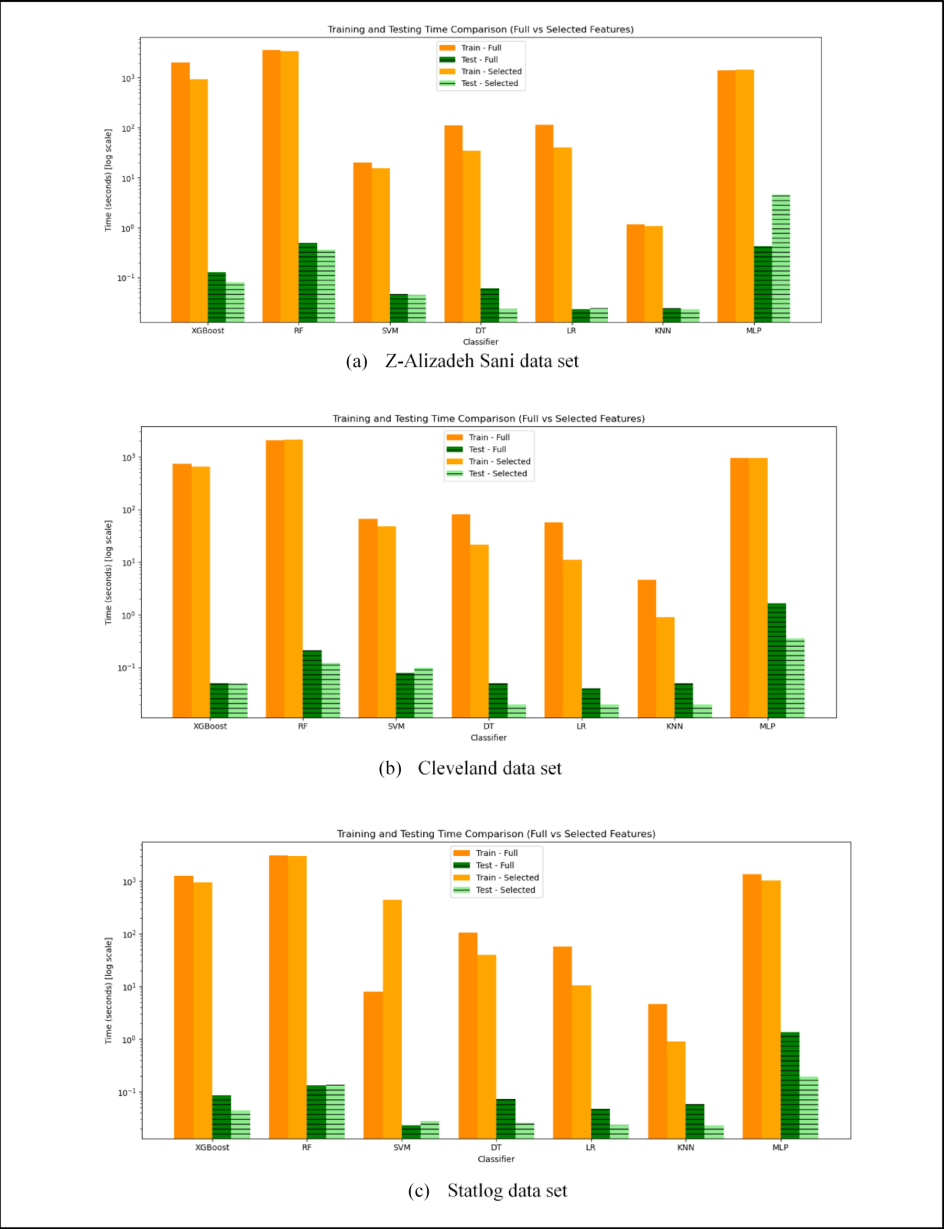
Training and testing time comparison of seven classifiers using all features and feature subsets selected by SHOW on (**a**) Z-Alizadeh Sani, (**b**) Cleveland, and (**c**) Statlog CAD data sets.

Beyond classification performance, evaluating the impact of feature selection on the computational efficiency of classification models is essential for assessing their practical applicability in CAD diagnosis systems. Therefore, the effect of the proposed SHOW-based feature selection on training and testing time is investigated. Figure 8 illustrates the comparison of training and testing times of all seven classifiers using the full feature set and the feature subsets selected by the SHOW algorithm across the three CAD data sets. As shown in the figure, employing the selected feature subsets generally leads to a noticeable reduction in both training and testing times compared to using the full feature set. This reduction is mainly attributed to the decreased dimensionality of the input feature space, which lowers the computational burden during model training and evaluation.

However, for certain classifiers, the training time obtained using the selected feature subsets is higher than that achieved with the full feature set. This behavior is primarily due to differences in the optimal hyperparameter configurations identified through grid-search tuning. In particular, the reduced feature space may result in more complex model structures, such as larger hidden layers or the adoption of more complex kernel functions, which can increase training time despite the reduced number of input features. Overall, the results demonstrate that the proposed SHOW-based feature selection effectively improves the computational efficiency of the classification models in terms of training and testing time while maintaining strong diagnostic performance, supporting its applicability in practical CAD diagnosis scenarios.

When analyzing the results on the Z-Alizadeh Sani data set, applying the proposed SHOW algorithm shows that the features Typical Chest Pain, Age, and Region RWMA are consistently selected across all classifiers, as shown in Table 13. These correspond to the first, third, and fourth features in Supplementary Table S8. Similarly, when examining the features selected on the Cleveland data set, the cp, ca, and thal features are selected by all classifiers, as indicated in Table 14, corresponding to the top three features in Table 10. On the other hand, the fbs feature, listed last in Table 10, is not selected by any classifier. For the Statlog data set, the cp, ca, and thal features are also selected by all classifiers, as shown in Table 15, and correspond to the first three features listed in Supplementary Table S10.

#### Comparison with state-of-the-art algorithms

Table 17 provides a comparison between the proposed SHOW algorithm and fourteen state-of-the-art feature selection methods reported in the literature. The results demonstrate that SHOW achieves a highly competitive balance between classification accuracy and the number of selected features across all considered data sets.

For the Z-Alizadeh Sani data set, SHOW achieves the highest reported accuracy of 93.79% using only 14 features, outperforming the N2Genetic-nuSVM method, which attained 93.08% accuracy while requiring 29 features (almost twice the number of features). This result highlights SHOW’s ability to effectively eliminate redundant and irrelevant features without compromising predictive performance. On the Cleveland data set, SHOW achieves an accuracy of 86.52% using only 5 selected features, surpassing methods such as RF-FSFC (86.14% with 8 features) and the brute-force approach (85.48% with 9 features). Similarly, for the Statlog data set, SHOW delivers the best reported accuracy of 87.78% with only 5 features, outperforming recent methods such as EEFS (85.55% with 6 features) and Hybrid PSO-EmNN (85.20% with 8 features).

These consistent results across multiple data sets highlight the effectiveness of the proposed SHOW algorithm, in which an ensemble SHAP-based feature ranking guides the optimized sequential forward selection wrapper technique, enabling strong diagnostic performance while avoiding exhaustive combinatorial evaluation of feature subsets.

**Table 17:** Performance comparison of the proposed algorithm against state-of-the-art research studies in terms of accuracy and number of selected features (in parentheses). Best results are shown in bold, and missing data is denoted by ‘–’.

Study, Year	Algorithm	Data set		
		Z-Alizadeh Sani	Cleveland	Statlog
^37^, 2019	N2Genetic-nuSVM	93.08 (29)	–	–
^42^, 2019	Brute force method	–	85.48 (9)	–
^38^, 2020	2HFS	92.58 (28)	–	–
^39^, 2020	RTs	91.47 (40)	–	–
^40^, 2020	Hybrid PSO-EmNN	88.34 (22)	84.00 (7)	85.20 (8)
^41^, 2022	QBSO-FS-SVM	90.10 (24)	84.40 (6)	84.80 (7)
^45^, 2022	MTE-RF	–	–	85.19 (8)
^43^, 2023	RF-FSFC	–	86.14 (8)	–
^44^, 2023	SVM-CFS/IG/SU	–	85.50 (10)	–
^46^, 2023	HRFLC	–	–	79.00 (11)
^3^, 2023	EEFS	91.78 (21)	85.47 (9)	85.55 (6)
	PEFS	91.14 (25)	–	–
^4^, 2024	PCA+AdaBoostM1	91.80 (5)	–	–
^47^, 2025	bAPO	–	–	83.15 (5)
^48^, 2025	SPFS	84.51 (3)	–	–
Proposed	SHOW	**93.79 (14)**	**86.52 (5)**	**87.78 (5)**

## Conclusions

In this study, we propose the SHOW feature selection algorithm, a novel two-stage approach that integrates the SHAP technique and an Optimized Wrapper method to improve the diagnosis of CAD. SHAP ranks the features according to their importance across three classifiers. It then applies an optimized sequential forward selection technique.

Extensive experimental results obtained on three benchmark data sets—Z-Alizadeh Sani, Cleveland, and Statlog—highlight the effectiveness of the proposed SHOW algorithm in reducing features while improving classification accuracy. Specifically, SHOW outperforms 14 state-of-the-art feature selection techniques: N2Genetic-nuSVM, Brute force, 2HFS, RTs, Hybrid PSO-EmNN, QBSO-FS-SVM, MTE-RF, RF-FSFC, SVM-CFS/IG/SU, HRFLC, EEFS, PCA+AdaBoostM1, bAPO, and SPFS. For example, using the XGBoost classifier, the proposed algorithm selected only 14 features (out of 55) from the Z-Alizadeh Sani data set, achieving an accuracy of 93.79% and a sensitivity of 93.98%; 5 features (out of 13) for the Cleveland data set, resulting in an accuracy of 86.52%, and a sensitivity of 88.55%; and 5 features (out of 13) for the Statlog data set, yielding an accuracy of 87.78%, and a sensitivity of 80%.

While the proposed algorithm demonstrates strong predictive performance, its high computational cost may limit its scalability on large or high-dimensional data sets. This is mainly attributed to the SHAP-based feature ranking stage, which requires computing feature attributions across all instances, as well as the wrapper-based feature selection process that involves repeated model training and evaluation as features are incrementally added. Future extensions of this work may involve evaluating the proposed approach on larger and more diverse CAD data sets to further enhance its generalizability and scalability. In addition, efficiency-improvement strategies, such as feature pre-filtering, as well as incorporating parallel or distributed computing techniques, can be explored to improve computational efficiency.

## Supplementary Information

Below is the link to the electronic supplementary material.

Supplementary Material 1.pdf includes detailed results that support the main findings of the study. Supplementary Figures S1–S4 show SHAP beeswarm and feature importance plots for the Z-Alizadeh Sani and Statlog data sets using the SHAP-XGBoost, SHAP-RF, and SHAP-SVM methods. Supplementary Tables S1–S10 present comprehensive feature importance scores, feature rank tables (FRT), and average rank tables (ART) generated from the three SHAP-based ranking approaches for both data sets.

## Data Availability

The CAD data sets used to support the results of this study are publicly available from the UCI Machine Learning Repository and can be accessed through the following links:- The Z-Alizadeh Sani data set at (https://archive.ics.uci.edu/dataset/412/z+alizadeh+sani).- The Cleveland data set at (https://archive.ics.uci.edu/dataset/45/heart+disease).- The Statlog data set at (https://archive.ics.uci.edu/dataset/145/statlog+heart).
