# Supplementary material for: An explainable AI-driven hybrid feature selection approach for coronary artery disease diagnosis
Source: Sci Rep. 2026 Mar 25;16:10411. doi: 10.1038/s41598-026-41712-y (PMC13031509; doi:10.1038/s41598-026-41712-y)
Supplement: Supplementary file 1 — Supplementary Material 1.pdf includes detailed results that support the main findings of the study. Supplementary Figures S1–S4 show SHAP beeswarm and feature importance plots for the Z-Alizadeh Sani and Statlog data sets using the SHAP-XGBoost, SHAP-RF, and SHAP-SVM methods. Supplementary Tables S1–S10 present comprehensive feature importance scores, feature rank tables (FRT), and average rank tables (ART) generated from the three SHAP-based ranking approaches for both data sets. [file 41598_2026_41712_MOESM1_ESM.pdf]

# An Explainable AI-Driven Hybrid Feature Selection Approach for Coronary Artery Disease Diagnosis

Tarneem Elemam, Hosam Refaat, Mohamed Makhlouf

## Supplementary Figures

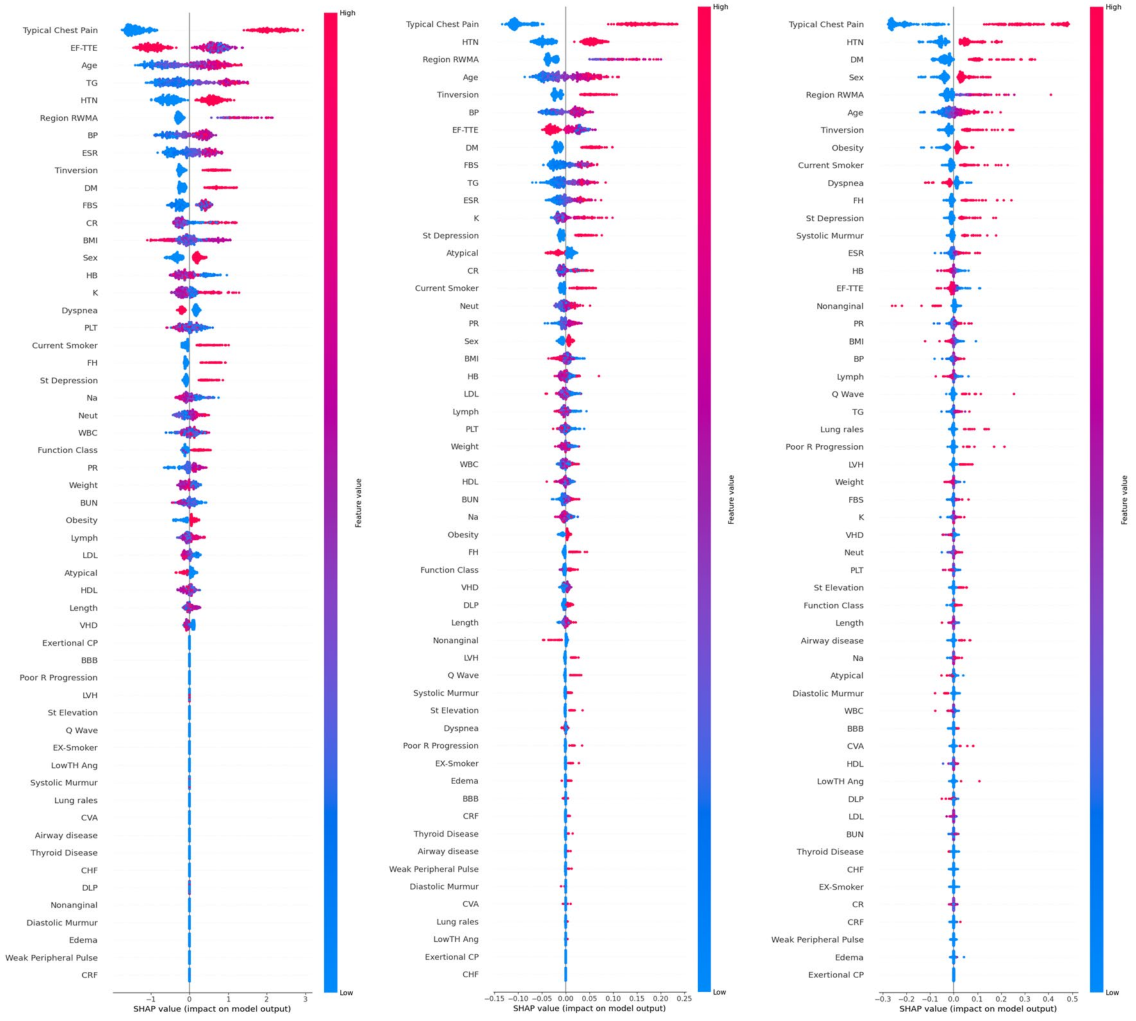


(a) (b) (c)

**Fig. S1.** SHAP beeswarm plots for the Z-Alizadeh Sani data set using three feature ranking methods: (a) SHAP-XGBoost, (b) SHAP-RF, (c) SHAP-SVM. The y-axis lists the features, and each dot corresponds to one instance. The position of each dot on the x-axis reflects the SHAP value for that instance, capturing both the direction and magnitude of the feature's contribution to the prediction, while the color reflects the feature value (red = high, blue = low).

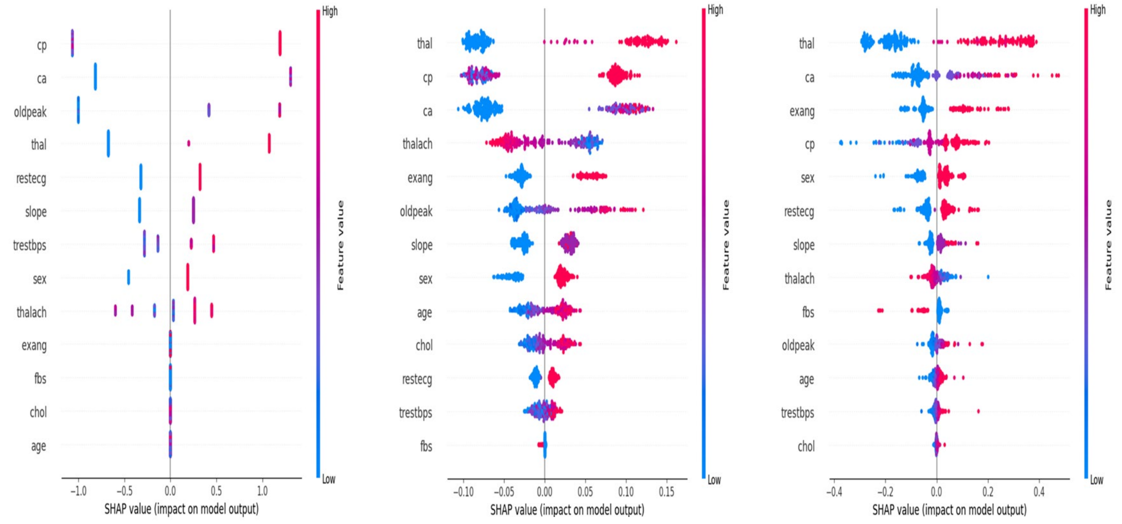


(a) (b) (c)

**Fig. S2.** SHAP beeswarm plots for the Statlog data set using three feature ranking methods: (a) SHAP-XGBoost, (b) SHAP-RF, (c) SHAP-SVM. The y-axis lists the features, and each dot corresponds to one instance. The position of each dot on the x-axis reflects the SHAP value for that instance, capturing both the direction and magnitude of the feature's contribution to the prediction, while the color reflects the feature value (red = high, blue = low).

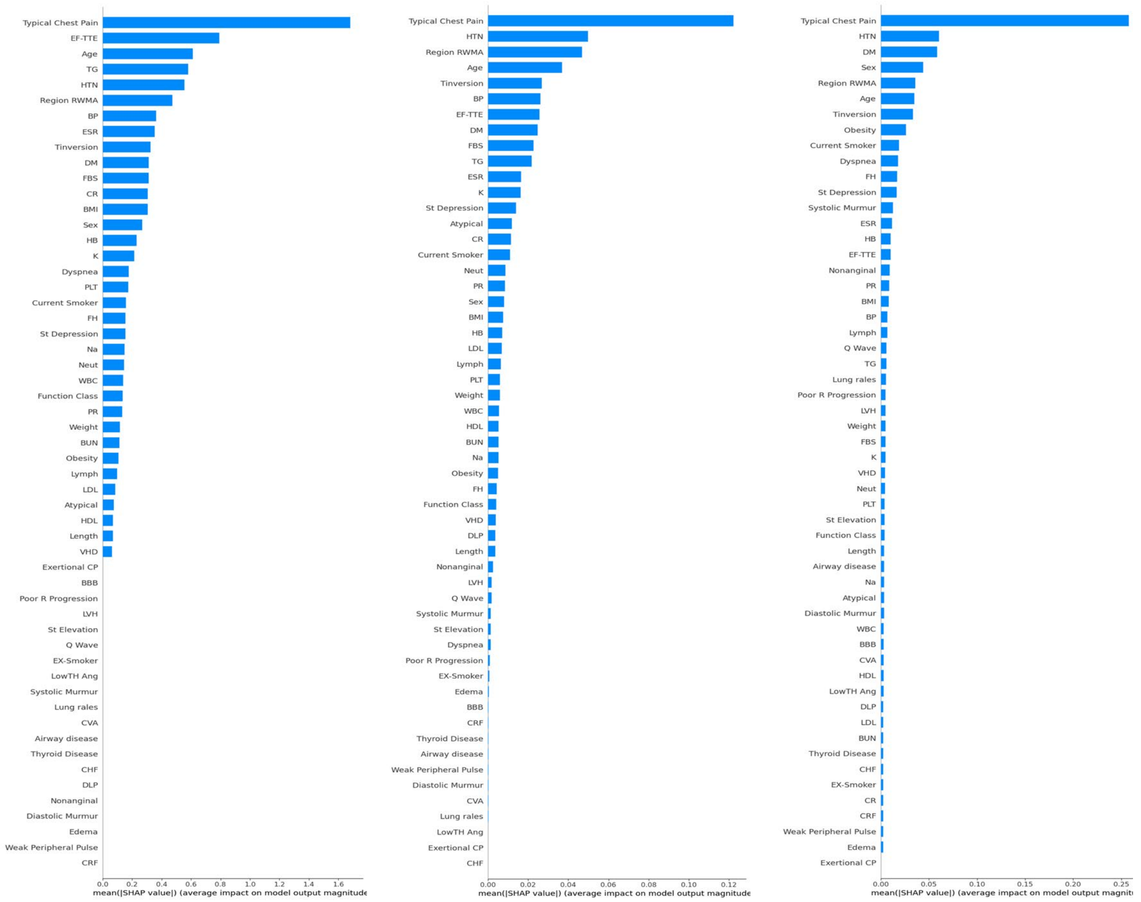


(a) (b) (c)

**Fig. S3.** Feature importance bar plots for the Z-Alizadeh Sani dataset, evaluated using three SHAP-based feature ranking methods: (a) SHAP-XGBoost, (b) SHAP-RF, and (c) SHAP-SVM. Each bar represents the relative importance of individual features in predicting outcomes. The height of each bar reflects the average absolute SHAP values of that feature across all instances, highlighting its average impact on the model.

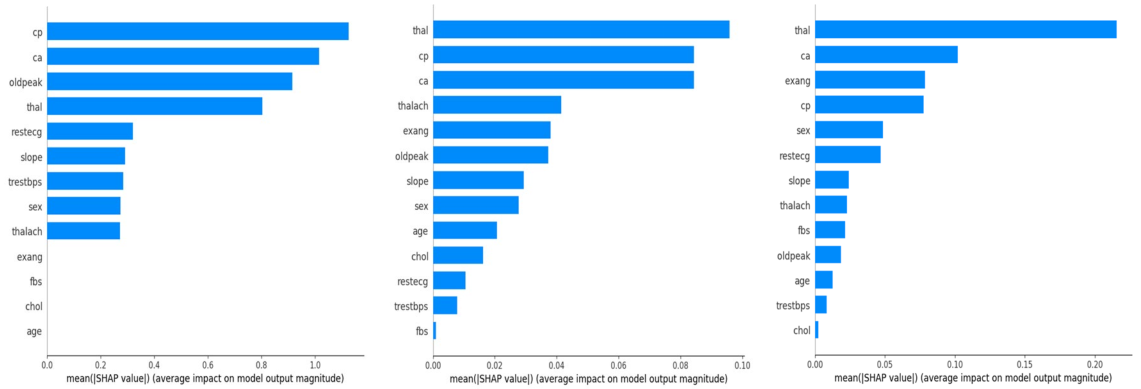


(a) (b) (c)

**Fig. S4.** Feature importance bar plots for the Statlog dataset, evaluated using three SHAP-based feature ranking methods: (a) SHAP-XGBoost, (b) SHAP-RF, and (c) SHAP-SVM. Each bar represents the relative importance of individual features in predicting outcomes. The height of each bar reflects the average absolute SHAP values of that feature across all instances, highlighting its average impact on the model.

# Supplementary Tables

**Table S1.** Feature importance scores for the Z-Alizadeh Sani dataset using SHAP-XGBoost method.

| Feature | Feature score | Feature rank |
|---|---|---|
| Typical Chest Pain | 1.682063 | 1 |
| EF-TTE | 0.792253 | 2 |
| Age | 0.612191 | 3 |
| TG | 0.580225 | 4 |
| HTN | 0.556663 | 5 |
| Region RWMA | 0.473825 | 6 |
| BP | 0.361887 | 7 |
| ESR | 0.353315 | 8 |
| Tinversion | 0.326470 | 9 |
| DM | 0.311873 | 10 |
| FBS | 0.311480 | 11 |
| CR | 0.306874 | 12 |
| BMI | 0.306206 | 13 |
| Sex | 0.268826 | 14 |
| HB | 0.231821 | 15 |
| K | 0.214471 | 16 |
| Dyspnea | 0.177469 | 17 |
| PLT | 0.174880 | 18 |
| Current Smoker | 0.158761 | 19 |
| FH | 0.155296 | 20 |
| St Depression | 0.153468 | 21 |
| Na | 0.146943 | 22 |
| Neut | 0.145056 | 23 |
| WBC | 0.140358 | 24 |
| Function Class | 0.136691 | 25 |
| PR | 0.132524 | 26 |
| Weight | 0.115671 | 27 |
| BUN | 0.112434 | 28 |
| Obesity | 0.107595 | 29 |
| Lymph | 0.097375 | 30 |
| LDL | 0.085302 | 31 |
| Atypical | 0.075419 | 32 |
| HDL | 0.069750 | 33 |
| Length | 0.067926 | 34 |
| VHD | 0.062102 | 35 |
| EX-Smoker | 0.000000 | 36 |
| CRF | 0.000000 | 37 |
| CVA | 0.000000 | 38 |
| Airway disease | 0.000000 | 39 |
| Thyroid Disease | 0.000000 | 40 |
| CHF | 0.000000 | 41 |
| DLP | 0.000000 | 42 |
| Edema | 0.000000 | 43 |
| Weak Peripheral Pulse | 0.000000 | 44 |
| Lung rales | 0.000000 | 45 |
| Systolic Murmur | 0.000000 | 46 |
| Diastolic Murmur | 0.000000 | 47 |
| Nonanginal | 0.000000 | 48 |
| Exertional CP | 0.000000 | 49 |
| LowTH Ang | 0.000000 | 50 |
| Q Wave | 0.000000 | 51 |
| St Elevation | 0.000000 | 52 |
| LVH | 0.000000 | 53 |
| Poor R Progression | 0.000000 | 54 |
| BBB | 0.000000 | 55 |

**Table S2.** Feature importance scores for the Z-Alizadeh Sani dataset using SHAP-RF method.

| Feature | Feature score | Feature rank |
|---|---|---|
| Typical Chest Pain | 0.122344 | 1 |
| HTN | 0.049738 | 2 |
| Region RWMA | 0.046759 | 3 |
| Age | 0.037026 | 4 |
| Tinversion | 0.026906 | 5 |
| BP | 0.026213 | 6 |
| EF-TTE | 0.025779 | 7 |
| DM | 0.024854 | 8 |
| FBS | 0.022749 | 9 |
| TG | 0.021701 | 10 |
| ESR | 0.016537 | 11 |
| K | 0.016180 | 12 |
| St Depression | 0.014016 | 13 |
| Atypical | 0.011925 | 14 |
| CR | 0.011399 | 15 |
| Current Smoker | 0.010936 | 16 |
| Neut | 0.008751 | 17 |
| PR | 0.008486 | 18 |
| Sex | 0.007986 | 19 |
| BMI | 0.007531 | 20 |
| HB | 0.007113 | 21 |
| LDL | 0.006986 | 22 |
| Lymph | 0.006315 | 23 |
| PLT | 0.005989 | 24 |
| Weight | 0.005862 | 25 |
| WBC | 0.005528 | 26 |
| HDL | 0.005335 | 27 |
| BUN | 0.005308 | 28 |
| Na | 0.005197 | 29 |
| Obesity | 0.005012 | 30 |
| FH | 0.004450 | 31 |
| Function Class | 0.004109 | 32 |
| VHD | 0.003960 | 33 |
| DLP | 0.003695 | 34 |
| Length | 0.003586 | 35 |
| Nonanginal | 0.002617 | 36 |
| LVH | 0.001956 | 37 |
| Q Wave | 0.001839 | 38 |
| Systolic Murmur | 0.001391 | 39 |
| St Elevation | 0.001333 | 40 |
| Dyspnea | 0.001316 | 41 |
| Poor R Progression | 0.001028 | 42 |
| EX-Smoker | 0.000732 | 43 |
| Edema | 0.000442 | 44 |
| BBB | 0.000333 | 45 |
| CRF | 0.000285 | 46 |
| Thyroid Disease | 0.000259 | 47 |
| Airway disease | 0.000254 | 48 |
| Weak Peripheral Pulse | 0.000229 | 49 |
| Diastolic Murmur | 0.000180 | 50 |
| CVA | 0.000151 | 51 |
| Lung rales | 0.000147 | 52 |
| LowTH Ang | 0.000084 | 53 |
| CHF | 0.000000 | 54 |
| Exertional CP | 0.000000 | 55 |

**Table S3.** Feature importance scores for the Z-Alizadeh Sani dataset using SHAP-SVM method.

| Feature | Feature score | Feature rank |
|---|---|---|
| Typical Chest Pain | 0.258061 | 1 |
| HTN | 0.060637 | 2 |
| DM | 0.058666 | 3 |
| Sex | 0.044034 | 4 |
| Region RWMA | 0.035610 | 5 |
| Age | 0.034705 | 6 |
| Tinversion | 0.033504 | 7 |
| Obesity | 0.026126 | 8 |
| Current Smoker | 0.019052 | 9 |
| Dyspnea | 0.018017 | 10 |
| FH | 0.016860 | 11 |
| St Depression | 0.016400 | 12 |
| Systolic Murmur | 0.012542 | 13 |
| ESR | 0.011516 | 14 |
| HB | 0.010377 | 15 |
| EF-TTE | 0.010300 | 16 |
| Nonanginal | 0.009117 | 17 |
| PR | 0.008574 | 18 |
| BMI | 0.008152 | 19 |
| BP | 0.006901 | 20 |
| Lymph | 0.006573 | 21 |
| Q Wave | 0.005999 | 22 |
| TG | 0.005835 | 23 |
| Lung rales | 0.005328 | 24 |
| Poor R Progression | 0.004988 | 25 |
| LVH | 0.004915 | 26 |
| Weight | 0.004906 | 27 |
| FBS | 0.004904 | 28 |
| K | 0.004658 | 29 |
| VHD | 0.004581 | 30 |
| Neut | 0.004441 | 31 |
| PLT | 0.003879 | 32 |
| St Elevation | 0.003742 | 33 |
| Function Class | 0.003690 | 34 |
| Length | 0.003639 | 35 |
| Airway disease | 0.003539 | 36 |
| Na | 0.003527 | 37 |
| Atypical | 0.003284 | 38 |
| Diastolic Murmur | 0.003161 | 39 |
| WBC | 0.003145 | 40 |
| BBB | 0.002949 | 41 |
| CVA | 0.002918 | 42 |
| HDL | 0.002897 | 43 |
| LowTH Ang | 0.002721 | 44 |
| DLP | 0.002624 | 45 |
| LDL | 0.002578 | 46 |
| BUN | 0.002575 | 47 |
| Thyroid Disease | 0.002505 | 48 |
| CHF | 0.002361 | 49 |
| EX-Smoker | 0.002348 | 50 |
| CR | 0.002287 | 51 |
| CRF | 0.002278 | 52 |
| Weak Peripheral Pulse | 0.002271 | 53 |
| Edema | 0.002264 | 54 |
| Exertional CP | 0.000000 | 55 |

**Table S4.** Feature importance scores for the Statlog dataset using SHAP-XGBoost method.

| Feature | Feature score | Feature rank |
|---|---|---|
| cp | 1.125763 | 1 |
| ca | 1.015835 | 2 |
| oldpeak | 0.915492 | 3 |
| thal | 0.803361 | 4 |
| restecg | 0.321128 | 5 |
| slope | 0.291413 | 6 |
| trestbps | 0.285211 | 7 |
| sex | 0.274016 | 8 |
| thalach | 0.272164 | 9 |
| age | 0.000000 | 10 |
| chol | 0.000000 | 11 |
| fbs | 0.000000 | 12 |
| exang | 0.000000 | 13 |

**Table S5.** Feature importance scores for the Statlog dataset using SHAP-RF method.

| Feature | Feature score | Feature rank |
|---|---|---|
| thal | 0.095886 | 1 |
| cp | 0.084417 | 2 |
| ca | 0.084335 | 3 |
| thalach | 0.041419 | 4 |
| exang | 0.038036 | 5 |
| oldpeak | 0.037272 | 6 |
| slope | 0.029318 | 7 |
| sex | 0.027753 | 8 |
| age | 0.020740 | 9 |
| chol | 0.016122 | 10 |
| restecg | 0.010498 | 11 |
| trestbps | 0.007839 | 12 |
| fbs | 0.000962 | 13 |

**Table S6.** Feature importance scores for the Statlog dataset using SHAP-SVM method.

| Feature | Feature score | Feature rank |
|---|---|---|
| thal | 0.215667 | 1 |
| ca | 0.102207 | 2 |
| exang | 0.078704 | 3 |
| cp | 0.077554 | 4 |
| sex | 0.048467 | 5 |
| restecg | 0.047002 | 6 |
| slope | 0.024294 | 7 |
| thalach | 0.022815 | 8 |
| fbs | 0.021620 | 9 |
| oldpeak | 0.018503 | 10 |
| age | 0.012725 | 11 |
| trestbps | 0.008298 | 12 |
| chol | 0.002483 | 13 |

**Table S7.** Feature rank table (FRT) for the Z-Alizadeh Sani dataset.

| Feature | SHAP-XGBoost | SHAP-RF | SHAP-SVM |
|---|---|---|---|
| Age | 3 | 4 | 6 |
| Weight | 27 | 25 | 27 |
| Length | 34 | 35 | 35 |
| Sex | 14 | 19 | 4 |
| BMI | 13 | 20 | 19 |
| DM | 10 | 8 | 3 |
| HTN | 5 | 2 | 2 |
| Current Smoker | 19 | 16 | 9 |
| EX-Smoker | 36 | 43 | 50 |
| FH | 20 | 31 | 11 |
| Obesity | 29 | 30 | 8 |
| CRF | 37 | 46 | 52 |
| CVA | 38 | 51 | 42 |
| Airway disease | 39 | 48 | 36 |
| Thyroid Disease | 40 | 47 | 48 |
| CHF | 41 | 54 | 49 |
| DLP | 42 | 34 | 45 |
| BP | 7 | 6 | 20 |
| PR | 26 | 18 | 18 |
| Edema | 43 | 44 | 54 |
| Weak Peripheral Pulse | 44 | 49 | 53 |
| Lung rales | 45 | 52 | 24 |
| Systolic Murmur | 46 | 39 | 13 |
| Diastolic Murmur | 47 | 50 | 39 |
| Typical Chest Pain | 1 | 1 | 1 |
| Dyspnea | 17 | 41 | 10 |
| Function Class | 25 | 32 | 34 |
| Atypical | 32 | 14 | 38 |
| Nonanginal | 48 | 36 | 17 |
| Exertional CP | 49 | 55 | 55 |
| LowTH Ang | 50 | 53 | 44 |
| Q Wave | 51 | 38 | 22 |
| St Elevation | 52 | 40 | 33 |
| St Depression | 21 | 13 | 12 |
| Tinversion | 9 | 5 | 7 |
| LVH | 53 | 37 | 26 |
| Poor R Progression | 54 | 42 | 25 |
| BBB | 55 | 45 | 41 |
| FBS | 11 | 9 | 28 |
| CR | 12 | 15 | 51 |
| TG | 4 | 10 | 23 |
| LDL | 31 | 22 | 46 |
| HDL | 33 | 27 | 43 |
| BUN | 28 | 28 | 47 |
| ESR | 8 | 11 | 14 |
| HB | 15 | 21 | 15 |
| K | 16 | 12 | 29 |
| Na | 22 | 29 | 37 |
| WBC | 24 | 26 | 40 |
| Lymph | 30 | 23 | 21 |
| Neut | 23 | 17 | 31 |
| PLT | 18 | 24 | 32 |
| EF-TTE | 2 | 7 | 16 |
| Region RWMA | 6 | 3 | 5 |
| VHD | 35 | 33 | 30 |

**Table S8.** Average rank table (ART) for the Z-Alizadeh Sani dataset.

| Feature | SHAP-XGBoost | SHAP-RF | SHAP-SVM | Avg |
|---|---|---|---|---|
| Typical Chest Pain | 1 | 1 | 1 | 1.000000 |
| HTN | 5 | 2 | 2 | 3.000000 |
| Age | 3 | 4 | 6 | 4.333333 |
| Region RWMA | 6 | 3 | 5 | 4.666667 |
| DM | 10 | 8 | 3 | 7.000000 |
| Tinversion | 9 | 5 | 7 | 7.000000 |
| EF-TTE | 2 | 7 | 16 | 8.333333 |
| BP | 7 | 6 | 20 | 11.000000 |
| ESR | 8 | 11 | 14 | 11.000000 |
| Sex | 14 | 19 | 4 | 12.333333 |
| TG | 4 | 10 | 23 | 12.333333 |
| Current Smoker | 19 | 16 | 9 | 14.666667 |
| St Depression | 21 | 13 | 12 | 15.333333 |
| FBS | 11 | 9 | 28 | 16.000000 |
| HB | 15 | 21 | 15 | 17.000000 |
| BMI | 13 | 20 | 19 | 17.333333 |
| K | 16 | 12 | 29 | 19.000000 |
| PR | 26 | 18 | 18 | 20.666667 |
| FH | 20 | 31 | 11 | 20.666667 |
| Obesity | 29 | 30 | 8 | 22.333333 |
| Dyspnea | 17 | 41 | 10 | 22.666667 |
| Neut | 23 | 17 | 31 | 23.666667 |
| PLT | 18 | 24 | 32 | 24.666667 |
| Lymph | 30 | 23 | 21 | 24.666667 |
| CR | 12 | 15 | 51 | 26.000000 |
| Weight | 27 | 25 | 27 | 26.333333 |
| Atypical | 32 | 14 | 38 | 28.000000 |
| Na | 22 | 29 | 37 | 29.333333 |
| WBC | 24 | 26 | 40 | 30.000000 |
| Function Class | 25 | 32 | 34 | 30.333333 |
| VHD | 35 | 33 | 30 | 32.666667 |
| Systolic Murmur | 46 | 39 | 13 | 32.666667 |
| LDL | 31 | 22 | 46 | 33.000000 |
| Nonanginal | 48 | 36 | 17 | 33.666667 |
| BUN | 28 | 28 | 47 | 34.333333 |
| HDL | 33 | 27 | 43 | 34.333333 |
| Length | 34 | 35 | 35 | 34.666667 |
| Q Wave | 51 | 38 | 22 | 37.000000 |
| LVH | 53 | 37 | 26 | 38.666667 |
| DLP | 42 | 34 | 45 | 40.333333 |
| Poor R Progression | 54 | 42 | 25 | 40.333333 |
| Lung rales | 45 | 52 | 24 | 40.333333 |
| Airway disease | 39 | 48 | 36 | 41.000000 |
| St Elevation | 52 | 40 | 33 | 41.666667 |
| EX-Smoker | 36 | 43 | 50 | 43.000000 |
| CVA | 38 | 51 | 42 | 43.666667 |
| Thyroid Disease | 40 | 47 | 48 | 45.000000 |
| CRF | 37 | 46 | 52 | 45.000000 |
| Diastolic Murmur | 47 | 50 | 39 | 45.333333 |
| BBB | 55 | 45 | 41 | 47.000000 |
| Edema | 43 | 44 | 54 | 47.000000 |
| CHF | 41 | 54 | 49 | 48.000000 |
| Weak Peripheral Pulse | 44 | 49 | 53 | 48.666667 |
| LowTH Ang | 50 | 53 | 44 | 49.000000 |
| Exertional CP | 49 | 55 | 55 | 53.000000 |

**Table S9.** Feature rank table (FRT) for the Statlog dataset.

| Feature | SHAP-XGBoost | SHAP-RF | SHAP-SVM |
|---|---|---|---|
| age | 10 | 9 | 11 |
| sex | 8 | 8 | 5 |
| cp | 1 | 2 | 4 |
| trestbps | 7 | 12 | 12 |
| chol | 11 | 10 | 13 |
| fbs | 12 | 13 | 9 |
| restecg | 5 | 11 | 6 |
| thalach | 9 | 4 | 8 |
| exang | 13 | 5 | 3 |
| oldpeak | 3 | 6 | 10 |
| slope | 6 | 7 | 7 |
| ca | 2 | 3 | 2 |
| thal | 4 | 1 | 1 |

**Table S10.** Average rank table (ART) for the Statlog dataset.

| Feature | SHAP-XGBoost | SHAP-RF | SHAP-SVM | Avg |
|---|---|---|---|---|
| thal | 4 | 1 | 1 | 2.000000 |
| cp | 1 | 2 | 4 | 2.333333 |
| ca | 2 | 3 | 2 | 2.333333 |
| oldpeak | 3 | 6 | 10 | 6.333333 |
| slope | 6 | 7 | 7 | 6.666667 |
| sex | 8 | 8 | 5 | 7.000000 |
| thalach | 9 | 4 | 8 | 7.000000 |
| exang | 13 | 5 | 3 | 7.000000 |
| restecg | 5 | 11 | 6 | 7.333333 |
| age | 10 | 9 | 11 | 10.000000 |
| trestbps | 7 | 12 | 12 | 10.333333 |
| chol | 11 | 10 | 13 | 11.333333 |
| fbs | 12 | 13 | 9 | 11.333333 |
